# Modulating prospective memory and attentional control with high-definition transcranial current stimulation: Study protocol of a randomized, double-blind, and sham-controlled trial in healthy older adults

**DOI:** 10.1371/journal.pone.0289532

**Published:** 2023-08-07

**Authors:** Nadine Schmidt, Marta Menéndez-Granda, Ronya Münger, Thomas P. Reber, Ute J. Bayen, Fabian E. Gümüsdagli, Alexandra Hering, Emilie Joly-Burra, Matthias Kliegel, Jessica Peter

**Affiliations:** 1 University Hospital of Old Age Psychiatry and Psychotherapy, University of Bern, Bern, Switzerland; 2 Graduate School for Health Sciences, University of Bern, Bern, Switzerland; 3 Faculty of Psychology, Uni Distance Suisse, Brig, Switzerland; 4 Institute for Experimental Psychology, Heinrich-Heine-Universität Düsseldorf, Düsseldorf, Germany; 5 Department of Developmental Psychology, Tilburg School for Social and Behavioural Sciences, Tilburg University, Tilburg, The Netherlands; 6 Cognitive Aging Lab (CAL), Faculty of Psychology and Educational Sciences, University of Geneva, Geneva, Switzerland; 7 Centre for the Interdisciplinary Study of Gerontology and Vulnerability, University of Geneva, Geneva, Switzerland; 8 Swiss Centre of Expertise in Life Course Research, LIVES Centre, Lausanne and Geneva, Switzerland; Public Library of Science, UNITED STATES

## Abstract

The ability to remember future intentions (i.e., prospective memory) is influenced by attentional control. At the neuronal level, frontal and parietal brain regions have been related to attentional control and prospective memory. It is debated, however, whether more or less activity in these regions is beneficial for older adults’ performance. We will test that by systematically enhancing or inhibiting activity in these regions with anodal or cathodal high-definition transcranial direct current stimulation in older adults. We will include n = 105 healthy older volunteers (60–75 years of age) in a randomized, double-blind, sham-controlled, and parallel-group design. The participants will receive either cathodal, anodal, or sham high-definition transcranial direct current stimulation of the left or right inferior frontal gyrus, or the right superior parietal gyrus (1mA for 20 min). During and after stimulation, the participants will complete tasks of attentional control and prospective memory. The results of this study will clarify how frontal and parietal brain regions contribute to attentional control and prospective memory in older healthy adults. In addition, we will elucidate the relationship between attentional control and prospective memory in that age group. The study has been registered with ClinicalTrials.gov on the 12th of May 2021 (trial identifier: NCT04882527).

## Introduction

The ability to perform an intended action at a future point in time is referred to as prospective memory. How well we remember these intentions seems to depend on attentional control, which enables us to direct (or shift) attention and to inhibit responding to distractions [1, 2]. Attentional control predicts laboratory prospective memory across all ages [3–6]. At a neuronal level, there may be a link between attentional control and prospective memory as well since, for example, the inferior frontal gyrus as well as the superior parietal lobe were active during both tasks in younger and middle-aged adults (see [7] for a meta-analysis). There is an ongoing debate, however, whether increased or decreased activity in these brain regions is beneficial for older adults’ attentional control [8–12] or prospective memory [13–16]. In the context of ageing, understanding the differential role these areas have for both cognitive functions would be important. To this end, modulating activity in these areas during these tasks can provide dynamic insights. This may help to develop further current models of how they are involved in attentional control and prospective memory in healthy ageing and to predict what goes wrong in diseases. So far, activity in the left or right inferior frontal gyrus or the superior parietal lobe was modulated during attentional control tasks. It was found that activity in the right hemisphere was particularly relevant for attentional control in both younger and older adults [17–21] although excitatory protocols (e.g., anodal stimulation) did not necessarily led to performance gains. For prospective memory, increasing activity in the left superior parietal lobe improved younger adults’ prospective memory more than increasing activity in the right superior parietal lobe [22, 23]. It is not completely understood, however, whether this effect generalizes to older adults, and/or whether modulating other brain regions such as the left or right inferior frontal gyrus has similar effects on prospective memory.

To answer these questions, we will systematically enhance or inhibit activity in frontal or parietal brain regions in healthy older adults during attentional control and prospective memory tasks. We will modulate activity in three areas; that is, the right superior parietal lobe, the right inferior frontal lobe or the left inferior frontal lobe. In order to replicate previous studies, we will test whether attentional control will predict laboratory prospective memory. In addition, we will test whether attentional control will also predict naturalistic prospective memory.

We hypothesize that the right inferior frontal gyrus and the right superior parietal lobe will be important for laboratory prospective memory as well as for attentional control. Modulation of the left inferior frontal cortex, however, will only lead to an improvement in prospective memory since attentional control seems particularly associated with the right hemisphere. Finally, we hypothesize that attentional control will predict laboratory prospective memory rather than self-reports or naturalistic prospective memory. Laboratory tasks share a similar setting and rely on timely motor responses while self-reports or naturalistic prospective memory tasks do not. To control for any effect of mood or agitation on cognition, we will examine current mood as well as the heart rate of all participants.

## Materials and methods

**Fig 1:**
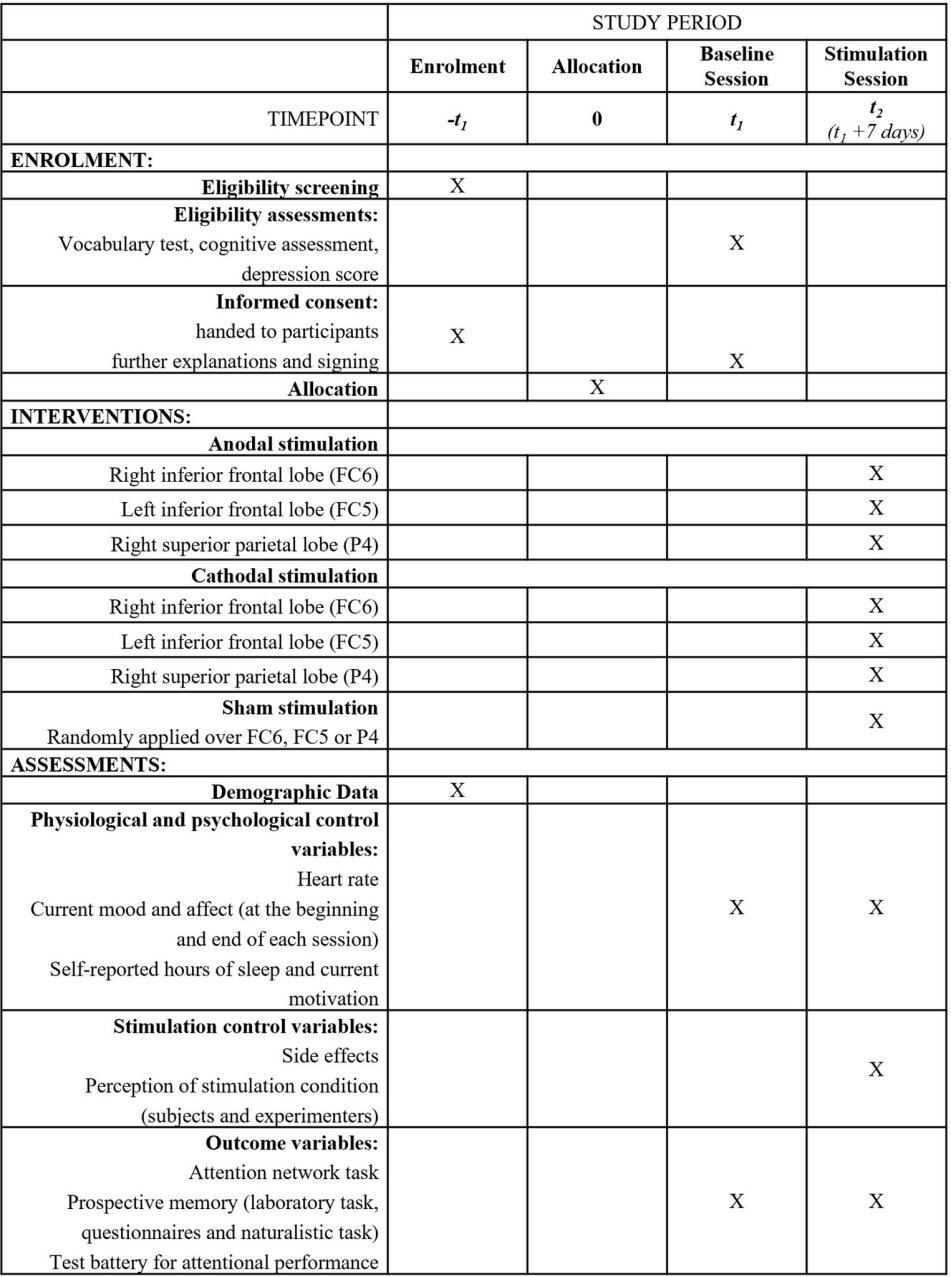
SPIRIT schedule of enrolment, interventions and assessments.

This study aims to test the contribution of frontal and parietal brain regions to attentional control and prospective memory in healthy older adults. We will conduct the study in a randomized, double-blind, sham-controlled, and parallel-group design (Fig 1). The study has been registered with ClinicalTrials.gov (Identifier: NCT04882527) and follows the SPIRIT guidelines [24].

### Participants’ eligibility and recruitment

Participants will be recruited via flyers, newspaper advertisement, or newsletters. For an inclusion, they will need to be between 60–75 years of age, fluent in German, right-handers, non-smokers, with normal or corrected-to-normal vision, and no history of severe psychiatric or neurological disorders. In addition, their Montreal Cognitive Assessment score (MoCA) [25] will need to be ≥ 23 and their Geriatric Depression Score (GDS) [26] will need to be ≤ 6. To ensure comparable verbal intelligence, all participants will complete a German vocabulary test (Wortschatztest, WST) [27]. Exclusion criteria will be severe colour blindness, past head injuries, dermatosis, metal implants in the head-area, current or lifetime seizures, alcohol or drug abuse, intake of medication that interferes with cognition, as well as brain damage.

All participants will give written informed consent prior to testing. The Cantonal Ethics Committee Bern approved the study, which will be conducted according to the Declaration of Helsinki.

### Randomization

A total of 105 healthy older adults will be included, stratified by age and sex. All participants will be randomly assigned to one of six experimental groups or the control condition (i.e., sham stimulation). They will receive anodal or cathodal high-definition tDCS of the right inferior frontal lobe, the left inferior frontal lobe, or the right superior parietal lobe, or sham. For sham stimulation, we will randomly place the electrodes over the same regions. Randomization will be done by JP based on a Matlab script written by TR (allocation ratio 1:5, stratified by age and sex). Neither JP nor TR will be involved in data collection. The study will follow a double-blind design, such that neither the participants nor the experimenter will be aware of the stimulation condition. Unblinding will occur at the end of data acquisition. Exceptions will only be allowed in case of adverse events.

### Study procedure

**Fig 2:**
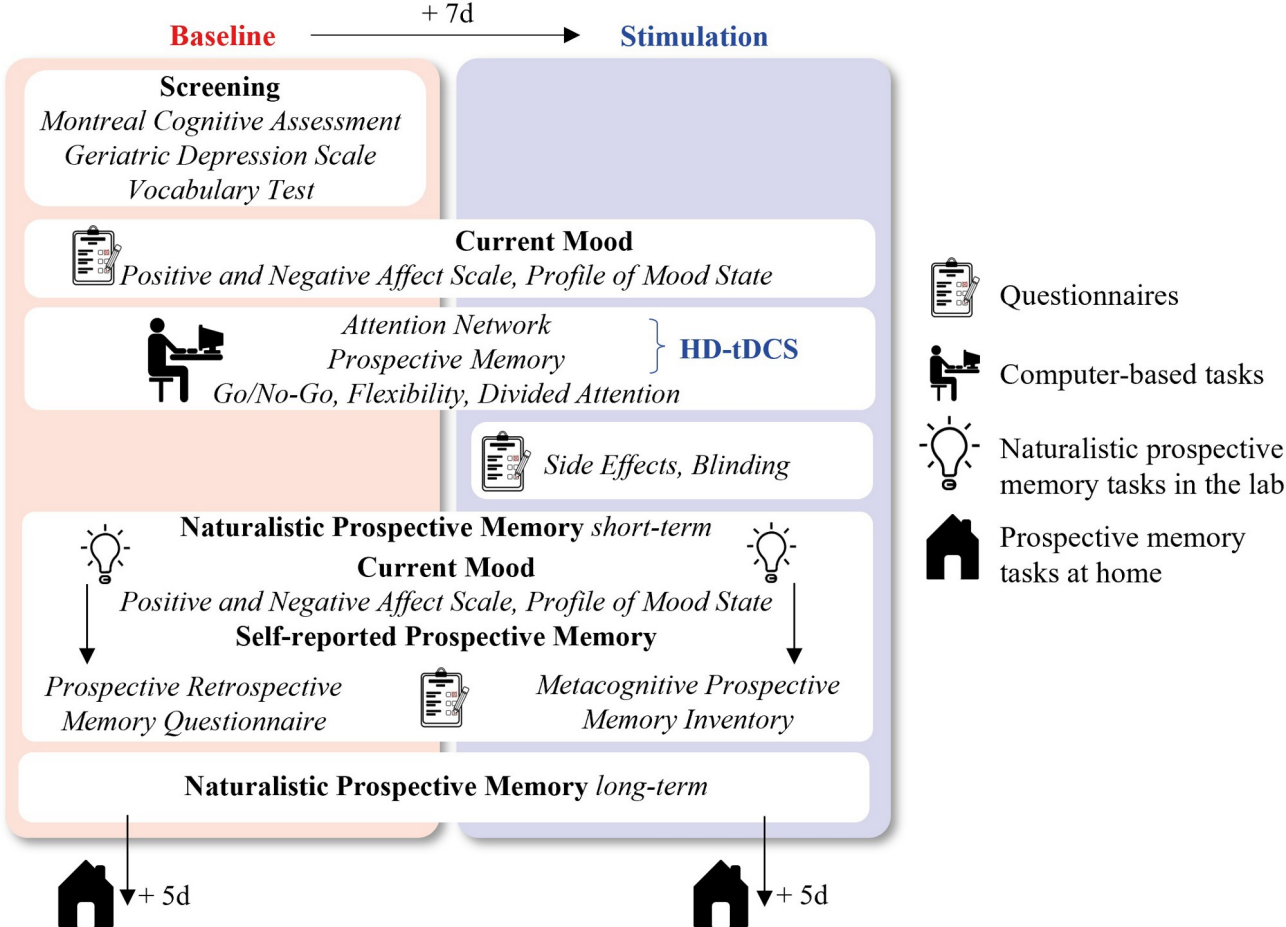
Study procedure. There will be two assessments with similar setups. Only during the second assessment, participants will receive high definition-transcranial direct current stimulation (HD-tDCS) for 20 minutes with 1 milliamp.

All participants will be tested twice to differentiate learning effects from stimulation effects (Fig 2). All assessments will be done by the same examiners (NS, RM) at the University Hospital of Old Age Psychiatry and Psychotherapy at the University of Bern, Switzerland. On the first day of the experiment, we will test verbal intelligence [27], followed by an assessment of current mood (Profile of Mood States, POMS; Positive and Negative Affect Scale, PANAS) [28–30]. Then, the participants will put on a wearable device (Polar Verity Sense by Polar Electro Europe AG, polar.ch) on their left arm that will monitor their heart rate. Next, they will be asked about how many hours they had slept the night before and how awake and motivated they currently feel. Then, the attention network task and the prospective memory task will follow. Next, they will do a Go/No-Go task, a flexibility task, and a divided attention task (TAP) [31]. Finally, they will again rate their current mood and different naturalistic prospective memory tasks will follow. The second day of the experiment will be identical with the exceptions that we will apply stimulation for 20 min during the attention network task and the prospective memory task and that there will be no verbal intelligence test. At the end of the experiment, side effects, and the participants’ perception of the stimulation condition will be captured [32].

### Attention network task (ANT)

**Fig 3:**
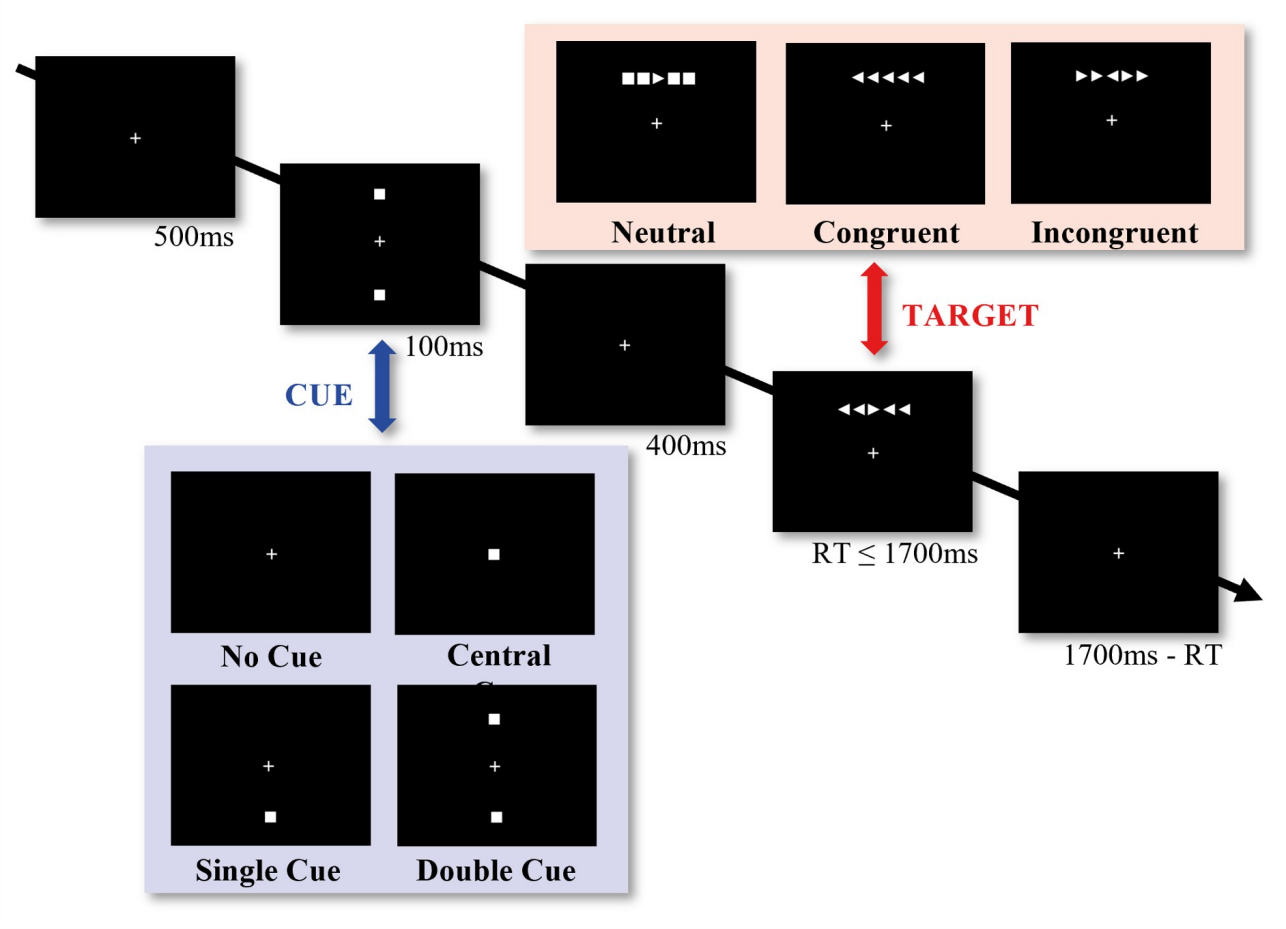
Attention network task. Participants will need to indicate the direction of a central arrow by button press. Flanking arrows (congruent or incongruent to the central arrow) or squares will need to be ignored. Arrows can be preceded by a cue or not. If a cue is present, it can appear at the centre (central cue) or both above and below the centre (double cue). It can also appear either above or below the fixation at which the arrow is going to appear (single cue).

With the attention network task, we will assess three aspects of attention: Alerting, orienting, and executive control [33]. We will use an adapted version of the original task (Fig 3) [34] and present stimuli with PsychoPy3 (version 2021.1.3). During the task, participants will see a row of five arrows on a screen and they will need to press one of two buttons to indicate the direction of the central arrow (i.e., left or right). The arrow can be surrounded by squares (neutral trials) or by flanker arrows that can point either to the same direction (congruent trials), or to the opposite direction (incongruent trials). The arrow can either appear above or below a fixation cross. A correct identification of the direction of any arrow requires attentional control since participants will need to inhibit responses to irrelevant flanking arrows and, at the same time, shift their attention either above or below any target location. In some trials, the arrow will be preceded by a cue. That cue can appear at the central fixation cross or both above and below it (i.e., double cue), which would indicate a neutral condition since no spatial cue will be provided. Sometimes, however, that cue will indicate where the next arrow will appear by either appearing below or above the central fixation (spatial cue condition). The task will consist of 144 trials with each combination of cue (no, double, middle, spatial) and stimuli (top neutral right/left, top congruent right/left, top incongruent right/left, bottom neutral right/left, bottom congruent right/left, bottom incongruent right/left) to be presented three times (4 cue types * 12 stimuli types * 3 repetitions = 144 trials). Instructions will be provided orally and in writing. All participants will train the task until they are able to provide a total of five correct responses. The training will be done immediately before the task starts.

We will assess alerting by calculating the difference between reaction times in trials with double cues and trials without any cues. For orienting, we will compare reaction times in trials with spatial cues to trials with central cues. For executive control (i.e., controlling), we will calculate the difference in response times between congruent flanker trials to incongruent flanker trials. The controlling variable will be of primary interest in our statistical analysis.

### Test battery for attentional performance (TAP)

The TAP is a computer-based test battery to assess attention [31]. We will use three different tasks from that battery to assess attentional control.

During the *Go/No-Go task*, participants will have to press a button whenever a go stimulus (i.e., a cross) will be shown on the screen. For no-go stimuli (i.e., a plus sign), a response needs to be inhibited.

During the *flexibility task*, a number and a letter are simultaneously presented left or right to a fixation cross. Participants will need to indicate the location of a target stimulus (i.e., either the number or the letter) with button presses. The target stimulus will alternate, beginning with the letter, then the number, then the letter and so on.

Finally, we will use a *dual task*. Participants will see different patterns of X’s on a grid. Whenever four X’s will build a square, the participants will need to press a button. Simultaneously, they will hear two alternating pitched tones. Whenever the same tone will be presented twice, the participants will need to press the same button.

For statistical analysis, we will calculate the number of false alarms during the Go/No-Go task, the number of correctly recognized targets during the flexibility task, and the number of missed targets during the dual task. For all three tasks, reaction times of correct answers will also be of primary interest. For secondary analyses, we will the intra-individual coefficient of variation (ICV), which examines attentional fluctuations [35].

### Laboratory prospective memory task

**Fig 4:**
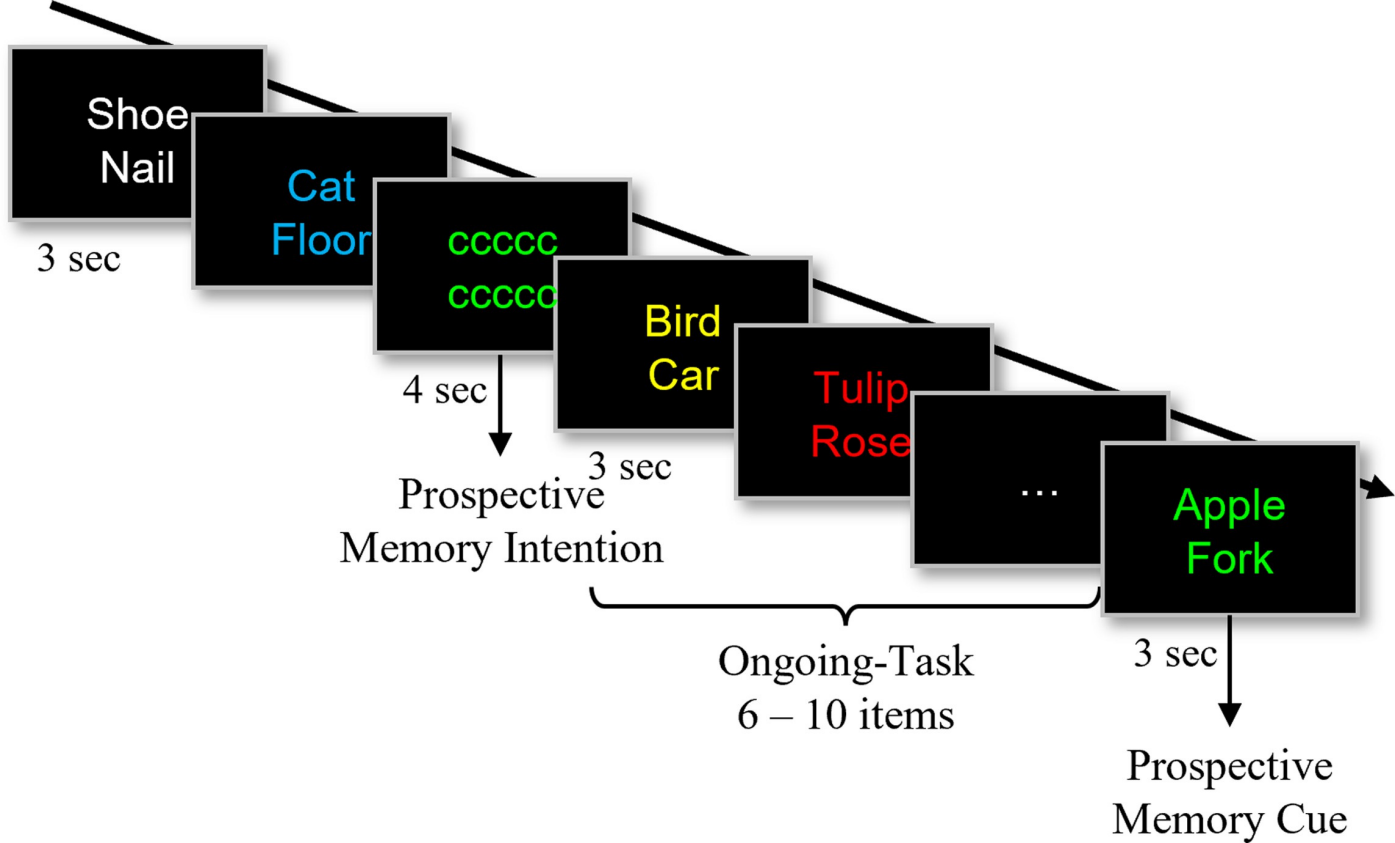
Prospective memory task. As an ongoing task, participants will need to indicate via button press whether two words belong to the same category or not. In addition, when a letter string appears, they will need to memorize the letter as well as its font colour (in the example ‘c’ and ‘green’). Whenever one of the following word pairs will be presented in the same colour as the letter string (here: green), participants will need to press the respective letter key (here: ‘c’).

We will use an adapted version of an event-based prospective memory task [36] and will again present stimuli with PsychoPy3 (v2021.1.3). The paradigm will consist of an ongoing task as well as a prospective memory task (Fig 4). During the ongoing task, the participants will indicate by button presses whether a word pair belongs to the same semantic category or not. For the prospective memory task, they will additionally need to remember the colour and the letter of a string of letters that will appear instead of a word pair from time to time (i.e., ‘v’ or ‘c’). Whenever one of the subsequent word pairs will be presented in the same colour as the letter string, participants will need to press the respective letter key (e.g., ‘c’; Fig 4). That means, they would need to inhibit a response to the ongoing task (i.e., indicating whether the two words belong to the same category or not). The letter string represents a prospective memory intention that has to be retrieved when a specific event occurs (i.e., event-based prospective memory). At the end of the task, an ongoing-only block will be added. This block will include prospective memory cues that the participants will need to ignore. With the ongoing-only block, we will assess possible aftereffects of prospective memory (i.e., difficulties to inhibit prospective memory intentions). The task will consist of 178 word pairs and 12 letter strings. The ongoing-only block will consist of 38 word pairs, and three (irrelevant) prospective memory letter strings. For the generation of word pairs, we will use a list of 633 words, from which 216 words (belonging to 36 different categories) [37, 38] will be chosen randomly (i.e., half of these will be used for each session). There will be two lists of words (A or B). Each list will contain the same words, either paired with another word from the same category or not. Half of the participants will see list A in the first session and list B in the second and vice versa. The letter strings (i.e., ‘v’ or ‘c’) will be presented for 4 s, either in magenta or in green. The word pairs for ongoing trials will be shown for 3 s in red, blue, white, yellow, cyan, or orange. Between a letter string and a word pair with the corresponding font colour (i.e., magenta or green), 6 to 10 word pairs will be presented. All ongoing-task colour fonts, except orange and cyan, may appear several times between the presentation of two letter strings. Cyan and orange will be used with similar frequency as magenta and green (i.e., prospective memory cue). This will control for effects of low frequency of colours: Words displayed in cyan or orange should be identified as ongoing-task stimuli although they appear equally often as the prospective memory cue colours. Again, task instructions will be provided orally and in writing. The participants will train the task with different word pairs until two prospective memory intentions will be correctly identified. Again, the training will be done immediately before the actual task begins.

For statistical analysis, we will focus on prospective memory accuracy and reaction times. Modulating brain activity might also influence performance in the ongoing task. Therefore, we will evaluate as a secondary analysis the mean reaction time of correct ongoing task trials (i.e., as an indicator of ongoing task performance) as well as their ICV, as an indicator of attentional fluctuations [35, 39, 40].

### Prospective memory questionnaires

We will use two different questionnaires to assess self-reported prospective and retrospective memory failures. In the first session, we will use the *Prospective Retrospective Memory Questionnaire* (PRMQ) [41]. With this questionnaire, the participants will need to rate how often they experience memory failures (16 examples) on a scale from 1 = very often to 5 = never. Half of the items refer to retrospective memory, the other half to prospective memory failures in everyday life. In the second session, we will use the *Metacognitive Prospective Memory Questionnaire* (MPMI-s) [42]. With this questionnaire we will assess prospective-memory abilities as well as strategy use for prospective remembering. The participants will rate 24 statements on a scale ranging from 1 = rarely to 5 = often.

### Naturalistic prospective memory tasks

To assess naturalistic prospective memory, we will adapt the *Royal Prince Alfred Prospective Memory Task* [43]. The task consists of two prospective intentions that need to be remembered during the on-site session as well as two intentions that need to be remembered later at home. We will use two different versions of the task, one for each session (see Table 1). The order of the versions will be counterbalanced across participants. The participants will receive a score in each of these tasks depending on how timely and accurately the intention was retrieved (ranging from 0 = late and incorrect retrieval to 3 = correct and timely retrieval). Participants will be told not to use any external memory aids. The naturalistic tasks will be as follows: The participants will need to alert the experimenter after finishing a specific questionnaire and when five minutes have elapsed. In addition, they will need to leave a message on the study phone as soon as they arrive at home as well as five days after the session. The participants will have to repeat the instructions to guarantee correct understanding. The sum of all scores achieved in the naturalistic prospective memory tasks will be used for statistical analysis.

**Table 1:** Naturalistic prospective memory tasks used in our study.

	Version A	Version B
**Short-term, event-based:**		
Between filling in the two questionnaires, please tell the experimenter…	to open the door to let some air in.	to get a new bottle of hand disinfectant.
**Short-term, time-based:**		
When five minutes have elapsed on the stopwatch, please tell the experimenter…	what you had for breakfast.	where you spent your last holiday.
**Long-term, event-based:**		
When you get home, please leave a message on the study phone about…	whether you are planning to watch TV this evening.	what you are planning to cook for dinner.
**Long-term, time-based:**		
In five days, please leave a message on the study phone about…	what the weather is like at your place.	what you are planning to do on this day.

### Heart rate

The participants will put on a wearable device (Polar Verity Sense by Polar Electro Europe AG, polar.ch) to their left arm with an elastic strap to monitor their heart rate. The device measures beats per minute for every second. Data will be saved on an internal memory during monitoring and will later be transferred to a computer. For statistical analysis we will use data assessed two minutes before the first task until two minutes after the prospective memory task (i.e., 24 min in total). Mean beats per minute within the first two minutes will serve as a baseline for each individual and will be used to standardize measurements of the following 22 minutes by subtracting the baseline from each of the following data points [44]. We will mainly assess the heart rate to control for a possible influence of agitation on cognition.

### Current mood and affect

The Profile of Mood States (POMS) [28, 29] and Positive and Negative Affect Scale (PANAS) [30] will be administered to measure participants current range of feelings. The PANAS consists of 20 terms that are rated on a 5-point scale regarding how strongly they currently experience the described feeling ranging from 1 = not at all to 5 = extremely. Half of the items are added up to a positive affect score, the other half to a negative affect score [30]. The items of the POMS are rated on a 7-point scale from 1 = not at all to 7 = extremely. The questionnaire consists of 35 items, which belong to the four different subscales “*depression*”, “*fatigue*”, “*displeasure*” and “*vigor*” [28, 29].

### High-definition transcranial direct current stimulation (HD-tDCS)

**Fig 5:**
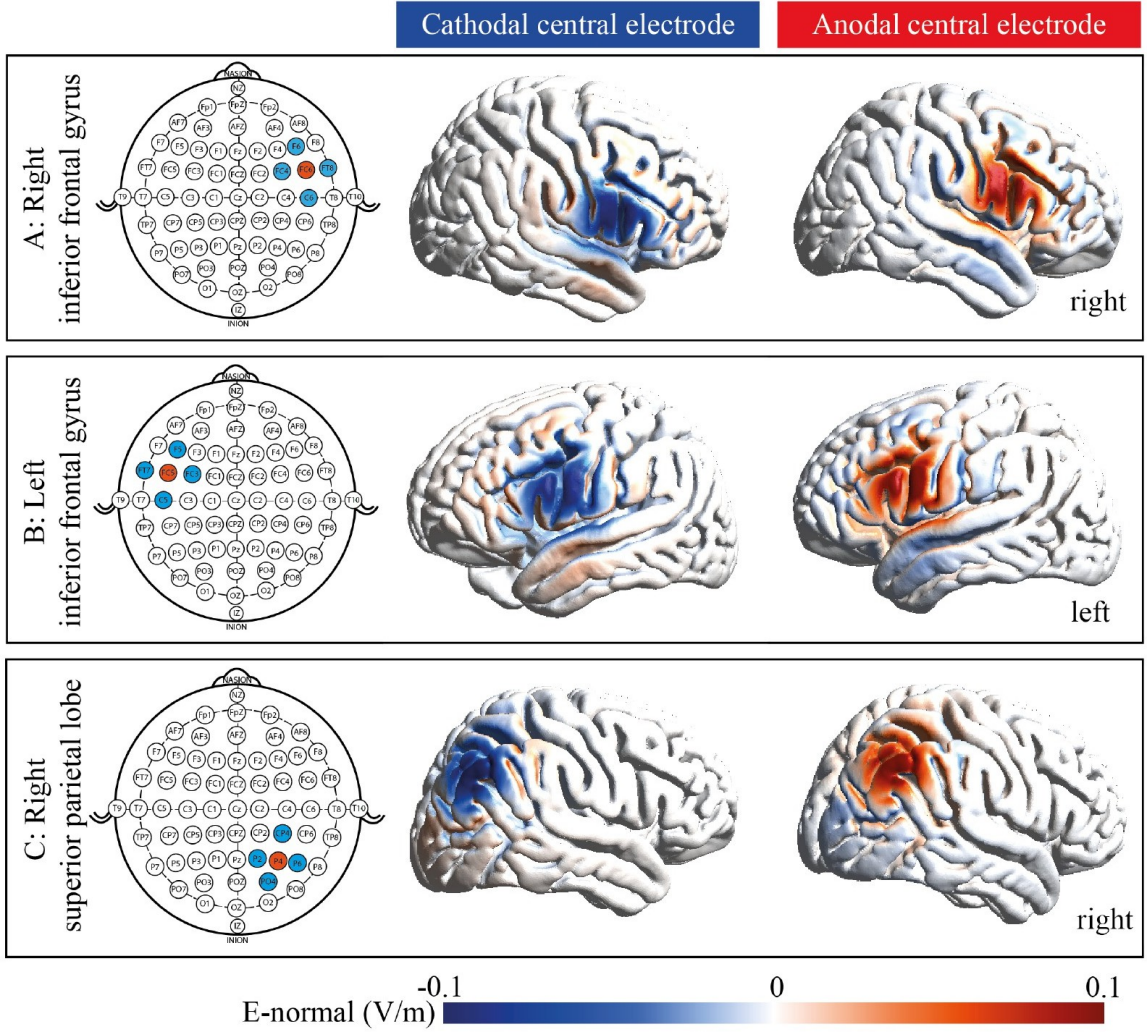
Electrode montage and current flow for high-definition transcranial direct current stimulation. We will modulate the right inferior frontal lobe (FC6; A), the left inferior frontal lobe (FC5; B), or the right superior parietal lobe (P4; C). Simulation illustrates current flow through the brain. Positive values (red) indicate an increase, negative values (blue) a decrease in excitability of neurons. Simulations were created with SimNIBS (version 3.2.6) [47].

We will use 4 x 1 ring electrodes for high-definition tDCS (Fig 5; Soterix Medical, NY, USA). One central electrode (anode or cathode) will be placed over the target brain region and four return electrodes will be placed around the central electrode in a ring-shape. This method is safe and well-tolerated with effective sham-control in older adults for up to 3 mA [45]. Total current density will be monitored by the device and will thus remain below safety limits [45]. For right or left inferior frontal gyrus stimulation, the central electrode will be placed over FC6 or FC5. For the right superior parietal gyrus, the central electrode will be placed over P4 according to the 10–20 system [46]. The adjoining electrodes will be placed approximately 3.5 cm away from the central electrode.

Real tDCS will consist of a 30 s ramp-up phase followed by constant current at 1 mA for 20 min and ramp-down for 30 s. For sham stimulation, the current will be ramped up to a current of 1 mA just as for real tDCS but it will be immediately ramped down again (in the beginning and at the end of 20 min). This sham procedure will produce similar sensations as real stimulation without exerting any stimulation effects [45, 48]. At the end of the experiment, side effects, and the participants’ perception of the stimulation condition will be captured [32]. We will regularly change the central electrode to prevent them from heavy and irregular abrasion [49, 50]. In addition, each set of electrodes will be used for 35 participants (i.e., each electrode will be used seven times as central and 28 times as a circle electrode). Lead quality of the electrodes will be measured after mounting of the electrodes, right before the start of the stimulation, and before demounting the electrodes. To ensure safety, the device will alarm and abort stimulation, if lead quality drops to a critical level. Should participants experience discomfort with the stimulation, it can be aborted at any point in time.

### Statistical analysis

Our primary outcome measures will be performance in attentional control and laboratory prospective memory tasks: From the ANT, we will evaluate the controlling variable. From the TAP, we will use the number of false alarms in the Go/No-Go tasks, correct answers in the flexibility task, the number of missed targets in the dual task, and mean reaction times. For laboratory prospective memory, accuracy and reaction times will be evaluated. We will use mixed ANOVAs or mixed effects models for statistical analysis of stimulation effects on primary outcome variables (depending on whether there will be any missing data). In contrast to an ANOVA, mixed effects models are better capable of handling missing data and they also allow an analysis of data on a trial level while taking hierarchical structures of data and variability within subjects into account. We will use session (first, second) as within-subject factor and condition (six experimental groups or sham) as between-subject factor. We will determine whether inhibiting or enhancing activity in the left or right frontal inferior lobe or the right superior parietal lobe will modulate attentional control or prospective memory.

As secondary outcomes, we will evaluate accuracy of the ongoing task as well as ICV of reaction times during attentional control or the ongoing-task of the laboratory prospective memory paradigm. In addition, we will test whether stimulation will have any effect on alerting or orienting (i.e., ANT Task). We will then test whether attentional control significantly predicts laboratory, naturalistic, or self-reported prospective memory using multiple linear regression.

Further we plan to model laboratory prospective memory responses by using multinomial modelling of response probabilities [51]. This approach estimates both the prospective component (i.e., remembering *that* you have to do something) and the retrospective component (e.g., remembering *when* and *what* you have to do) of prospective memory.

We will include age as a covariate in all models since it significantly influenced prospective memory in our previous study [52]. We will control for any potential influence of mood or agitation on cognition and test, in an exploratory analysis, whether mood or heart rate of participants will influence attentional control or prospective memory. Further we examine, whether one of these two measures are modulated by non-invasive brain stimulation. For an assessment of heart rate, we will include changes in beats per minute (relative to baseline) between the start of stimulation until two minutes after its end (i.e., 22 minutes). Generalized estimated equations will then be used to test any longitudinal stimulation effects on heart rate during the stimulation session [53–55].

We will use R (version 4.2.1) with RStudio (version 2022.02.3) for statistical analyses with *p* < 0.05 considered statistically significant. We will correct for multiple-comparisons using Bonferroni-Holm correction. Whenever the assumptions of normality and homogeneity are not met, data will be transformed and/or non-parametric alternatives will be used. We will only analyse data of participants that attended both sessions.

### Sample size calculation

We used G*power [56] for the determination of sample size. Calculations were based on a previous non-invasive brain stimulation study in healthy young participants [22]. The effect size in this study (*η*² = 0.32) suggested a required sample size of n = 93 to detect robust effects in an ANOVA design with seven groups. We have increased the sample size to n = 15 in each group and will, therefore, include n = 105 healthy older individuals.

### Data management

We will pseudonymise all study data (i.e., participants will be given a unique participant number). The coding key will be stored separately and locked away. Each participant will be informed orally and in writing about the nature, usage, and storage of their data. Data processing will be done on personal computers/laptops and institutional servers. All computers will be password-protected and encrypted. At the end of the study, all personal data will be deleted. The procedures comply with Swiss data privacy laws. Data will be saved in Dropbox folders encrypted with Boxcryptor or on Research Electronic Data Capture (REDCap), hosted by the Clinical Trials Unit (CTU) Bern. Paper pencil data will be stored in folders that are locked away. The study team will be responsible for data management; data monitoring will be done by an independent researcher not involved in the study. We will record any spontaneously reported adverse events or other unintended effects.

### Study status

Recruitment of participants started in May 2021. Data acquisition is still ongoing, and we expect to finish data acquisition in autumn 2023.

## Discussion

In the current study, we will systematically enhance or inhibit activity in brain regions known to be associated with attentional control and prospective memory. This may contribute to our understanding whether more or less activity in these regions is associated with better attentional control or prospective memory in older healthy adults. We expect that enhancing activity in these brain regions will lead to more accurate and faster responses in laboratory prospective memory and attentional control tasks, due to increased excitability of underlying neurons. In contrast, we expect opposite effects when inhibiting activity. There is some evidence to suggest, though, that increasing the excitability of neurons does not necessarily lead to an increase in cognitive performance. Complex interactions between stimulation type, duration, stimulation strength as well as task characteristics or mood of the participants may play a role [57]. It could therefore be that inhibiting activity in a given region may actually increase task performance, due to an improved signal-to-noise ratio [58]. The theory behind that is that when a stimulus in the environment elicits activity in a brain region, stimulus-irrelevant noise is also present. The higher the signal in comparison to the noise, the more accurate the response of the participant. If stimulation does not enhance the signal-noise ratio but rather reduces it, performance may drop.

Besides performance improvement or worsening, it could also be that we will find differential effects on outcome measures. An increase in accuracy, for example, could be linked to slower responses, or reduced accuracy could be linked to faster responses. Since we will use different attentional control tasks and both laboratory and naturalistic prospective memory tasks, we will be able to disentangle these interactions and discuss possible implications.

The results of our study will allow future perspectives for non-invasive brain stimulation research in the fields of prospective memory and attentional control in older adults. If attentional control or prospective memory can be enhanced with high-definition tDCS, its feasibility for long-term interventions may be evaluated in the future. A combination of cognitive training and repeated stimulation may, for instance, be promising. A recent meta-analysis by Jones and colleagues [59] found that a cognitive training with memory and/ or attention exercises can be useful for improving prospective memory. These effects may even be enhanced by non-invasive brain stimulation. As tDCS merely modulates brain activity that is already present (e.g., evoked by a task), the stimulation protocol may need to be personalized (e.g., using neuro-navigation) [60]. Compared to conventional tDCS, high-definition tDCS allows focal stimulation and also prevents bidirectional modulation within a brain area [61]. Therefore, predictions of stimulation effects on cognitive outcomes may be more precise and personalized stimulation may be more accurate. The transfer of training or stimulation effects from the laboratory to participants’ daily lives will be a big challenge for future interventions, though.

The results of our study, in particular the predictive value of attentional control for prospective memory, may contribute to this topic. Based on the integrative framework by Zuber and colleagues [2], we expect to find differences in the predictive value, depending on the ecological validity of the prospective memory task. The analysis of the predictive value may also contribute to a better understanding of the ‘age-prospective memory paradox’ which postulates that an impaired prospective memory in older adults is typically found in laboratory, but not in naturalistic tasks [62]. If we find evidence that attentional control is differentially predictive of naturalistic vs. laboratory tasks, this may contribute to the explanation of the paradox and may provide hints on how laboratory prospective memory tasks should be adapted to address similar functions as in naturalistic tasks.

High-definition tDCS is a relatively new method and therefore, only few studies have used it and have assessed feasibility, tolerability, and success of blinding in a sample of older adults (e.g., [45]). Our study will therefore add important insights in this age group. Finally, we will add to the literature whether stimulation has any effect on the heart rate of older healthy participants or on their current mood. Frontal brain regions contribute to the regulation of emotions and mood [63, 64], but also to the heart rate via the ‘brain-heart axis’ or the ‘frontal-vagal network’ [65]. A recent meta-analysis found small to medium effect sizes on the heart rate in 18 studies that stimulated frontal areas among others [66] but this meta-analysis only included one study with older adults suffering from stroke. Therefore, it is not well understood whether non-invasive brain stimulation has any effect on the heart rate in older healthy adults.

In sum, our study will systematically test the contribution of frontal and parietal brain regions to attentional control and prospective memory in older healthy adults. Our results may provide further insights how these cognitive functions are related on a behavioural and a neuronal level in that age group.

## Supporting information

S1 File SPIRIT checklist. (PDF)

S2 File Ethics application form. (PDF)

## List of abbreviations

ANT: Attention Network Task
HD-tDCS: high-definition transcranial Direct Current Stimulation
ICV: Intra-individual Coefficient of Variation
PANAS: Positive and Negative Affect Scale
POMS: Profile of Mood States
SPIRIT: Standard Protocol Items: Recommendations for Interventional Trials
TAP: Test battery for Attentional Performance
tDCS: transcranial Direct Current Stimulation
WST: Wortschatztest (German vocabulary test)
