# Supplementary material for: Modulating prospective memory and attentional control with high-definition transcranial current stimulation: Study protocol of a randomized, double-blind, and sham-controlled trial in healthy older adults
Source: PLoS One. 2023 Aug 7;18(8):e0289532. doi: 10.1371/journal.pone.0289532 (PMC10406223; doi:10.1371/journal.pone.0289532)
Supplement: S1 File — (PDF) [file pone.0289532.s001.pdf]

S1 File. SPIRIT 2013 Checklist*
populated *by the authors of*
“Modulating prospective memory and attentional control with high-definition transcranial current stimulation: study protocol of a randomized, double-blind, and sham-controlled trial in healthy older adults”

**Checklist retrieved from* [GUIDANCE FOR CLINICAL TRIAL PROTOCOLS (spirit-statement.org)](), June 8th, 2023

Section/item	Item No	Description
**Administrative information**		
Title	1	Descriptive title identifying the study design, population, interventions, and, if applicable, trial acronym<br>➔ *Title page (1)*
Trial registration	2a	Trial identifier and registry name. If not yet registered, name of intended registry<br>➔ *Abstract, “trial registration” (2) and Section “Materials and Methods” (4)*
	2b	All items from the World Health Organization Trial Registration Data Set<br>➔ *See “Financial disclosure statement”*
Protocol version	3	Date and version identifier<br>➔ n/a
Financial disclosure statement	4	Sources and types of financial, material, and other support<br>➔ *See “Financial disclosure statement”*
Roles and responsibilities	5a	Names, affiliations, and roles of protocol contributors<br>➔ *Title page (1) and Section “Authors contribution”*
	5b	Name and contact information for the trial sponsor<br>➔ *Title page (1)*
	5c	Role of study sponsor and funders, if any, in study design; collection, management, analysis, and interpretation of data; writing of the report; and the decision to submit the report for publication, including whether they will have ultimate authority over any of these activities<br>➔ *See “Financial disclosure statement”*

	5d	Composition, roles, and responsibilities of the coordinating centre, steering committee, endpoint adjudication committee, data management team, and other individuals or groups overseeing the trial, if applicable (see Item 21a for data monitoring committee)<br>➔ *Data management plan (14)*
**Introduction**		
Introduction and rationale	6a	Description of research question and justification for undertaking the trial, including summary of relevant studies (published and unpublished) examining benefits and harms for each intervention<br>➔ *Sections “Introduction” (2) and “High-definition transcranial direct current stimulation (HD-tDCS)” (11)*
	6b	Explanation for choice of comparators<br>➔ *Section “Introduction” (2)*
Objectives	7	Specific objectives or hypotheses<br>➔ *Section “Introduction” (2)*
Trial design	8	Description of trial design including type of trial (eg, parallel group, crossover, factorial, single group), allocation ratio, and framework (eg, superiority, equivalence, noninferiority, exploratory)<br>➔ *Sections “Materials and Methods” (4), «Randomization» (4) and “Study procedure” (5)*
**Methods: Participants, interventions, and outcomes**		
Study setting	9	Description of study settings (eg, community clinic, academic hospital) and list of countries where data will be collected. Reference to where list of study sites can be obtained<br>➔ *Section “Study procedure” (5)*
Eligibility criteria	10	Inclusion and exclusion criteria for participants. If applicable, eligibility criteria for study centres and individuals who will perform the interventions (eg, surgeons, psychotherapists)<br>➔ *Section “Participants eligibility and recruitment” (4)*
Interventions	11a	Interventions for each group with sufficient detail to allow replication, including how and when they will be administered<br>➔ *Sections “Study procedure” (5) and “High-definition transcranial direct current stimulation (HD-tDCS)” (11)*
	11b	Criteria for discontinuing or modifying allocated interventions for a given trial participant (eg, drug dose change in response to harms, participant request, or improving/worsening disease)<br>➔ *Section “High-definition transcranial direct current stimulation (HD-tDCS)” (11)*

	11c	Strategies to improve adherence to intervention protocols, and any procedures for monitoring adherence (eg, drug tablet return, laboratory tests)<br>➔ *n/a: stimulation is applied and monitored by experimenters in on-site session*
	11d	Relevant concomitant care and interventions that are permitted or prohibited during the trial<br>➔ *n/a: no restrictions*
Outcomes	12	Primary, secondary, and other outcomes, including the specific measurement variable (eg, systolic blood pressure), analysis metric (eg, change from baseline, final value, time to event), method of aggregation (eg, median, proportion), and time point for each outcome. Explanation of the clinical relevance of chosen efficacy and harm outcomes is strongly recommended<br>➔ *Sections "Study procedure" (5) to "Statistical Analysis" (12)*
Participant timeline	13	Time schedule of enrolment, interventions (including any run-ins and washouts), assessments, and visits for participants. A schematic diagram is highly recommended (see Figure)<br>➔ *Figure 1 "SPIRIT schedule" (4), Section "Study procedure" (5) including as well Figure 2 "Study procedure" (6)*
Sample size	14	Estimated number of participants needed to achieve study objectives and how it was determined, including clinical and statistical assumptions supporting any sample size calculations<br>➔ *Section "Sample size calculation" (14)*
Recruitment	15	Strategies for achieving adequate participant enrolment to reach target sample size<br>➔ *Section "Participants eligibility and recruitment" (4)*

Methods: Assignment of interventions (for controlled trials)

Allocation:

Sequence generation	16a	Method of generating the allocation sequence (eg, computer-generated random numbers), and list of any factors for stratification. To reduce predictability of a random sequence, details of any planned restriction (eg, blocking) should be provided in a separate document that is unavailable to those who enrol participants or assign interventions<br>➔ *Section «Randomization» (4)*
Allocation concealment mechanism	16b	Mechanism of implementing the allocation sequence (eg, central telephone; sequentially numbered, opaque, sealed envelopes), describing any steps to conceal the sequence until interventions are assigned<br>➔ *Section «Randomization» (4)*

Implementation	16c	Who will generate the allocation sequence, who will enrol participants, and who will assign participants to interventions<br>➔ *Section «Randomization» (4) and "Study procedure" (5)*
Blinding (masking)	17a	Who will be blinded after assignment to interventions (eg, trial participants, care providers, outcome assessors, data analysts), and how<br>➔ *Section «Randomization» (4) and "Study procedure" (5)*
	17b	If blinded, circumstances under which unblinding is permissible, and procedure for revealing a participant's allocated intervention during the trial<br>➔ *Section «Randomization» (4)*
**Methods: Data collection, management, and analysis**		
Data collection methods	18a	Plans for assessment and collection of outcome, baseline, and other trial data, including any related processes to promote data quality (eg, duplicate measurements, training of assessors) and a description of study instruments (eg, questionnaires, laboratory tests) along with their reliability and validity, if known. Reference to where data collection forms can be found, if not in the protocol<br>➔ *Sections "Study procedure" (5) to "Current mood and affect", "Statistical Analysis" (12) and "Data management" (14)*
	18b	Plans to promote participant retention and complete follow-up, including list of any outcome data to be collected for participants who discontinue or deviate from intervention protocols<br>➔ *n/a: no follow-up; in case of discontinuation we request consent to examine data assessed so far*
Data management	19	Plans for data entry, coding, security, and storage, including any related processes to promote data quality (eg, double data entry; range checks for data values). Reference to where details of data management procedures can be found, if not in the protocol<br>➔ *Section "Data management" (14)*
Statistical methods	20a	Statistical methods for analysing primary and secondary outcomes. Reference to where other details of the statistical analysis plan can be found, if not in the protocol<br>➔ *Section "Statistical Analysis" (12)*
	20b	Methods for any additional analyses (eg, subgroup and adjusted analyses)<br>➔ *Section "Statistical Analysis" (12)*
	20c	Definition of analysis population relating to protocol non-adherence (eg, as randomised analysis), and any statistical methods to handle missing data (eg, multiple imputation)<br>➔ *n/a*

Methods: Monitoring

Data monitoring	21a	Composition of data monitoring committee (DMC); summary of its role and reporting structure; statement of whether it is independent from the sponsor and competing interests; and reference to where further details about its charter can be found, if not in the protocol. Alternatively, an explanation of why a DMC is not needed<br>➔ *Section "Data management" (14)*
	21b	Description of any interim analyses and stopping guidelines, including who will have access to these interim results and make the final decision to terminate the trial<br>➔ *No interim analysis due to double blinding*
Harms	22	Plans for collecting, assessing, reporting, and managing solicited and spontaneously reported adverse events and other unintended effects of trial interventions or trial conduct<br>➔ *Section "Data management" (14)*
Auditing	23	Frequency and procedures for auditing trial conduct, if any, and whether the process will be independent from investigators and the sponsor<br>➔ *No audit trials*

Ethics and dissemination

Research ethics approval	24	Plans for seeking research ethics committee/institutional review board (REC/IRB) approval<br>➔ *See "Ethics approval and consent to participate"*
Protocol amendments	25	Plans for communicating important protocol modifications (eg, changes to eligibility criteria, outcomes, analyses) to relevant parties (eg, investigators, REC/IRBs, trial participants, trial registries, journals, regulators)<br>➔ *Only in case of severe adverse events (reporting to ethics committee)*
Consent or assent	26a	Who will obtain informed consent or assent from potential trial participants or authorised surrogates, and how (see Item 32)<br>➔ *See "Ethics approval and consent to participate"*
	26b	Additional consent provisions for collection and use of participant data and biological specimens in ancillary studies, if applicable<br>➔ *n/a*
Confidentiality	27	How personal information about potential and enrolled participants will be collected, shared, and maintained in order to protect confidentiality before, during, and after the trial<br>➔ *Section "Data management" (14)*

Declaration of interests	28	Financial and other competing interests for principal investigators for the overall trial and each study site<br>➔ *See "Competing interests"*
Access to data	29	Statement of who will have access to the final trial dataset, and disclosure of contractual agreements that limit such access for investigators<br>➔ *Section Availability of data and materials" (15)*
Ancillary and post-trial care	30	Provisions, if any, for ancillary and post-trial care, and for compensation to those who suffer harm from trial participation<br>➔ *n/a*
Dissemination policy	31a	Plans for investigators and sponsor to communicate trial results to participants, healthcare professionals, the public, and other relevant groups (eg, via publication, reporting in results databases, or other data sharing arrangements), including any publication restrictions<br>➔ *Section "Availability of data and materials" (15)*
	31b	Authorship eligibility guidelines and any intended use of professional writers<br>➔ *See "Authors' contribution"*
	31c	Plans, if any, for granting public access to the full protocol, participant-level dataset, and statistical code<br>➔ *Section "Availability of data and materials" (15)*
**Appendices**		
Informed consent materials	32	Model consent form and other related documentation given to participants and authorised surrogates<br>➔ *n/a (only available in German)*
Biological specimens	33	Plans for collection, laboratory evaluation, and storage of biological specimens for genetic or molecular analysis in the current trial and for future use in ancillary studies, if applicable<br>➔ *n/a*

*It is strongly recommended that this checklist be read in conjunction with the SPIRIT 2013 Explanation & Elaboration for important clarification on the items. Amendments to the protocol should be tracked and dated. The SPIRIT checklist is copyrighted by the SPIRIT Group under the Creative Commons "Attribution-NonCommercial-NoDerivs 3.0 Unported" license.

Reference: Chan A-W, Tetzlaff JM, Gotzsche PC, Altman DG, Mann H, Berlin JA, et al. SPIRIT 2013 explanation and elaboration: guidance for protocols of clinical trials. BMJ. 2013;346:e7586–e7586.
