# Supplementary material for: Modulating prospective memory and attentional control with high-definition transcranial current stimulation: Study protocol of a randomized, double-blind, and sham-controlled trial in healthy older adults
Source: PLoS One. 2023 Aug 7;18(8):e0289532. doi: 10.1371/journal.pone.0289532 (PMC10406223; doi:10.1371/journal.pone.0289532)
Supplement: S2 File — (PDF) [file pone.0289532.s002.pdf]

Modulating prospective memory in older adults with non-invasive brain stimulation

---

Title used in informed consent
Verbessert nicht-invasive Hirnstimulation das prospektive Gedächtnis bei älteren Menschen?

Study Type:	Clinical Trial with Medical Device (MD)
Risk Categorisation:	Risk category A
Study Registration:	The study will be registered with clinicaltrials.gov
Sponsor:	PD Dr. Jessica Peter<br>Universität Bern<br>Universitätsklinik für Alterpsychiatrie und Psychotherapie<br>Bolligenstrasse 111, 3000 Bern 60<br>Phone: +41 31 932 89 03
Investigator:	Prof. Dr. Stefan Klöppel<br>Universität Bern<br>Universitätsklinik für Alterpsychiatrie und Psychotherapie<br>Murtenstrasse 21, 3008 Bern<br>Phone: +41 31 632 88 17
Investigational product:	Soterix high-definition stimulator
Version and Date:	Version 1.3 (dated 2021-01-19)

CONFIDENTIALITY STATEMENT
The information contained in this document is confidential and the property of the sponsor. The information may not - in full or in part - be transmitted, reproduced, published, or disclosed to others than the applicable Competent Ethics Committee(s) and Regulatory Authority(ies) without prior written authorisation from the sponsor except to the extent necessary to obtain informed consent from those who will participate in the study.

PROTOCOL SIGNATURE FORM

Study Title	Modulating prospective memory in older adults via non-invasive brain stimulation

The Sponsor has approved the protocol version 1.3 (dated 2021-01-19) and confirms hereby to conduct the study according to the protocol, current version of the World Medical Association Declaration of Helsinki, and ICH-GCP guidelines as well as the local legally applicable requirements.

Sponsor:

Name: PD Dr. Jessica Peter

Date: __________________________ Signature: ____________________________

Principal Investigator:

Study Title	Modulating prospective memory in older adults via non-invasive brain stimulation

Principal Investigator:

Name: Prof. Dr. Stefan Klöppel

Date: ____________________________ Signature: ______________________________

TABLE OF CONTENTS

TABLE OF CONTENTS 3
GLOSSARY OF ABBREVATIONS 4
1 STUDY SYNOPSIS 5
2 BACKGROUND AND RATIONALE 7
3 STUDY OBJECTIVES AND DESIGN 8
3.1 Hypothesis and primary objective 8
3.2 Primary and secondary endpoints 9
3.3 Study design 9
3.4. Study intervention 10
4 STUDY POPULATION AND STUDY PROCEDURES 10
4.1 Inclusion and exclusion criteria, justification of study population 10
4.2 Recruitment, screening and informed consent procedure 11
4.3 Study procedures 11
Telephone screening 12
On-site visit 12
4.4 Withdrawal and discontinuation 14
5 STATISTICS AND METHODOLOGY 14
5.1. Statistical analysis plan and sample size calculation 14
5.2. Handling of missing data and drop-outs 15
6 SAFETY 15
6.1 Local regulations / Declaration of Helsinki 15
6.2 Medical Device Category A studies 15
6.7 (Premature) termination of study 15
6.8 Insurance 16
7 FURTHER ASPECTS 16
7.1 Overall ethical considerations 16
7.2 Risk-benefit assessment 16
8 QUALITY CONTROL AND DATA PROTECTION 16
8.1 Quality measures 16
8.2 Data recording and source data 16
8.3 Confidentiality and coding 16
8.4 Retention and destruction of study data and biological material 17
9 MONITORING AND REGISTRATION 17
10. FUNDING / PUBLICATION / DECLARATION OF INTEREST 18
10 REFERENCES 18
Appendix 1: Schedule of assessments 21

GLOSSARY OF ABBREVATIONS

AE .... Adverse Event
ASR/DSUR .... Annual Safety Report
BASEC .... Business Administration System for Ethical Committees
ClinO .... Ordinance on Clinical Trials in Human Research
CRF .... Case Report Form
CTU .... Clinical Trials Unit
DLPFC .... Dorsolateral Prefrontal Cortex
EDC .... Electronic Data Capturing
fMRI .... functional Magnetic Resonance Imaging
GCP .... Good Clinical Practice
GDS .... Geriatric Depression Score
HD-tDCS .... High-Definition Transcranial Direct Current Stimulation
HRA .... Human Research Act
ICH .... International Conference on Harmonisation
MoCA .... Montreal Cognitive Assessment
PM .... Prospective Memory
REDCap .... Research Electronic Data Capture
RPFC .... Rostral Prefrontal Cortex
RT .... Reaction time
SAE .... Serious Adverse Event
tDCS .... Transcranial Direct Current Stimulation
TMS .... Transcranial Magnetic Stimulation

1 STUDY SYNOPSIS

**Sponsor / Investigator**	Sponsor<br>PD Dr. Jessica Peter<br>University of Bern<br>University Clinic of Old Age Psychiatry and Psychotherapy<br>Bolligenstrasse 111, 3000 Bern 80<br>Phone: +41 31 932 89 03<br><br>Investigator<br>Prof. Dr. Stefan Klöppel<br>University of Bern<br>University Clinic of Old Age Psychiatry and Psychotherapy<br>Murtenstrasse 21, 3008 Bern<br>Phone: +41 31 632 88 17
**Study Title**	Modulating prospective memory in older adults via non-invasive brain stimulation
**Short Title / Study ID**	NA
**Protocol Version and Date**	Version 1.3 (2021-01-19)
**Study Registration**	The study will be registered with clinicaltrials.gov
**Study Category and Rationale**	Risk category A<br>Transcranial direct current stimulation is a non-invasive technique that will entail only minimal risks to the participants. Potential risk groups will be excluded from participation based on a prior telephone screening.
**Background and Rationale**	Prospective memory (PM) is the ability to remember to carry out intentions with a certain delay. PM tasks require a large degree of self-initiated retrieval and in the absence of a prompt to recall, people must 'remember to remember' by their own volition. Thus, PM is a challenge - especially in old age with increasing health-related PM demands. In healthy young adults, previous studies highlighted the involvement of frontal or parietal regions but whether these regions are similarly important for elderly adults' PM is still virtually unknown. Yet, the mere occurrence of a change in brain activity in concomitance with performance of a behavioral task is not sufficient to confirm a causal relationship between the two phenomena. A functional facilitation or inhibition of these areas via non-invasive brain stimulation will lead to clearer evidence that the neural activity in these areas is not only concomitant with the cognitive operations involved in the behavioral task but actually underlies them. Therefore, our study aims to apply non-invasive brain stimulation to facilitate or inhibit activity in frontal or parietal regions presumed to be functionally associated with PM. We will implement control tasks (i.e., attentional control) to assess whether stimulation will specifically enhance PM performance or whether it will transfer to other cognitive functions. In addition, we will implement a baseline assessment of both the PM task and the control task to disentangle learning effects from stimulation effects.

**Risk / Benefit Assessment**	Transcranial direct current stimulation (tDCS) entail only minimal risks provided the exclusion of potential risk groups (e.g., individuals with magnetisable implants etc.). We will ensure the exclusion of such risk groups via prior telephone screening. The participants will be reimbursed for participation with 50 CHF.
**Objective(s)**	With non-invasive brain stimulation, we will identify if increased or decreased activity in left or right left frontal or right parietal regions will enhance PM performance in elderly adults and whether the modulation of performance via tDCS will be specific for PM.
**Endpoint(s)**	We will focus on the modulation of accuracy and reaction times via tDCS (both in the control tasks and in the PM tasks).
**Study Design**	We will apply a double blind, sham-controlled, parallel group design. We will randomly assign the participants to one of seven groups (cathodal vs. anodal right inferior cortex; cathodal vs. anodal left inferior frontal gyrus; cathodal vs. anodal right superior parietal cortex; or sham).
**Statistical Considerations**	We will compare the effect of stimulation on performance in PM (both laboratory and real-life) as well as attentional control with ANOVAs using stimulation group as between-subject factor and session as a within-subject factor. We will focus on non-parametric alternatives to ANOVA for reaction times. We will correct for multiple comparisons, using the Bonferroni-Holm procedure where appropriate.
**Inclusion- / Exclusion Criteria**	All participants should be between 60-80 years of age, fluent in German, non-smokers, with normal or corrected-to-normal vision and no history of severe psychiatric or neurological disorders. Further exclusion criteria will be past head injuries, dermatosis, metal implants in the head-area, seizures, current or lifetime alcohol or drug abuse, intake of psychotropic drugs, brain damage, as well as magnetisable implants (e.g., cardiac pacemakers, brain stimulator).<br>Participants will be included if their Montreal Cognitive Assessment (MoCA) score is ≥ 26 and their Geriatric Depression Score (GDS) is ≤ 6. To ensure comparable verbal intelligence scores, participants will complete a German vocabulary test (Wortschatztest, WST) during the on-site visit. All participants will give written informed consent prior to testing.
**Number of Participants with Rationale**	We conducted a sample size calculation using g*power. Since there is currently no published tDCS study in PM, we based calculations on a previous TMS study in healthy young individuals, applying event-based PM. The effect size in this study (η^2= 0.32) suggests a required sample size of n=93 to detect robust effects in an ANOVA fixed-effects design with seven groups (i.e., cathodal and anodal stimulation of the right and left inferior frontal gyrus, cathodal and anodal stimulation to the right superior parietal cortex, and sham stimulation). We will increase the sample size to n=15 in each group and will, therefore, include n=105 healthy elderly individuals in this project.
**Study Intervention**	We will use 4x1 ring electrodes for high-definition transcranial direct current stimulation (HD-tDCS). In this approach, a center ring electrode (anode or cathode) overlying the target cortical region is surrounded by four return electrodes, which help circumscribe the area of stimulation. Real tDCS will be applied with an intensity of 1 mA. The stimulator will be operated in 'study mode', such that neither the participant nor the examiner will be aware of the experimental condition (i.e., double-blind study). The codes for sham and real tDCS will be provided by REDCap via CTU Bern.
**Control Intervention**	The control intervention is sham tDCS. For sham tDCS, the electrical current will be ramped up and switched off after 30 s. This procedure is known to elicit the same sensations (notably itching/ tingling) as real tDCS without eliciting stimulation effects.
**Study procedures**	We will randomly assign the participants to one of seven groups (cathodal or anodal stimulation of the left inferior frontal gyrus, the right inferior frontal gyrus, the right superior parietal cortex, or sham stimulation). We will apply a baseline session prior

	to the stimulation session in which we will assess both primary and secondary outcome measures. This will allow to disentangle learning effects from stimulation effects. During both sessions, the participants will first be asked to report the hours of sleep during the last night and to rate the quality of sleep as well as their current concentration (on a scale ranging from 0 to 10). Then, attentional control tasks and the PM task will be conducted. For the stimulation session, HD-tDCS will be applied for 20 minutes during which the participants will perform the attentional control task and the PM task. We implemented the control tasks to evaluate whether stimulation will particularly enhance PM or whether it will transfer to other cognitive functions. At the end of the experiment, we will we enquire about perceived side effects of the stimulation and control the consistent blinding of the participants with respect to the stimulus condition. During each session and the days afterwards, participants will be asked to perform naturalistic PM tasks. This will allow investigate whether stimulation only modulates laboratory PM or whether it will also modulate more realistic PM tasks.
**Study Duration and Schedule**	4 years, November 2019 – December 2023<br>Month Year of First-Participant-In (planned): February 2021<br>Month Year of Last-Participant-Out (planned): August 2023
**Investigator(s)**	Prof. Dr. Stefan Klöppel (Principal Investigator)<br>University of Bern<br>University Clinic of Old Age Psychiatry and Psychotherapy<br>Murtenstrasse 21, 3008 Bern<br>Phone: +41 31 632 88 17<br>Mail: stefan.kloeppel@upd.unibe.ch<br><br>PD Dr. Jessica Peter (Sponsor)<br>University of Bern<br>University Clinic of Old Age Psychiatry and Psychotherapy<br>Bolligenstrasse 111, 3000 Bern 80<br>Phone: +41 31 932 89 03<br>Mail: Jessica.peter@upd.unibe.ch<br><br>Nadine Schmidt (PhD student)<br>University of Bern<br>University Clinic of Old Age Psychiatry and Psychotherapy<br>Bolligenstrasse 111, 3000 Bern 80<br>Tel. +41 (0)31 930 93 21<br>Mail: nadine.schmidt@upd.unibe.ch
**Study Center(s)**	Monocentric study and the University Clinic of Old Age Psychiatry and Psychotherapy, University of Bern
**GCP Statement**	This study will be conducted in compliance with the protocol, the current version of the Declaration of Helsinki, the ICH-GCP, the HRA as well as other locally relevant legal and regulatory requirements.

2 BACKGROUND AND RATIONALE

Prospective memory (PM) refers to the ability to remember to carry out future intentions at either a certain time (i.e., time-based PM), following an event (i.e., a specific external cue; event-based PM), or upon the completion of another task (i.e., activity-based PM) (1). PM tasks are critical to

a wide-range of everyday activities and they are particularly important in aging, since forgetting intentions such as meeting a doctor's appointment, taking medication, or turning of the oven after cooking could be life threatening. Thus, proper PM abilities in older age are of key relevance for maintaining functional independence and well-being (2). Consequently, there is growing interest in evaluating new strategies to counteract PM decline in aging, with the challenge on how to modulate PM to achieve the best possible behavioural outcome. PM comprises multiple phases that rely on different cognitive processes. First, a person needs to form an intention (e.g., call a fried at six o'clock in the evening). During this intention-encoding phase, the person plans when (i.e., at six o'clock) and how (i.e., by phone) the intention will be performed. Then, the intention is stored in retrospective memory (i.e., memory for past events, plans, or intentions), while the person is engaged in other activities (i.e., ongoing activity). When the moment for completing the intention arises, the person has to retrieve the intended action, inhibit other ongoing activities (in case of event-based and time-based PM tasks), switch to the intention, and perform it as planned (3). Thus, PM tasks are characterized by involving a delay between forming an intention and carrying it out, by including both an ongoing and a PM task, and by self-initiated retrieval of the delayed intention.

One critical cognitive component of PM tasks is strategic monitoring, which is influenced by attentional control (4–6). In addition, at least in younger adults, similar brain areas were found to be active during PM tasks and attentional control tasks (6–10). Moreover, age-related changes in neuronal activity during PM tasks seem to mirror those of attentional control tasks. However, there is an ongoing debate as to whether activity during these tasks in older adults is enhanced or reduced compared to younger adults: Peira et al. (2016) reported that older adults exhibited reduced activity in the left and right inferior frontal gyri during a PM task. Moreover, activity in the left inferior frontal gyrus correlated negatively with response time during PM tasks, indicating faster retrieval processes in older adults (11). On the contrary, Gonneaud et al. (2011) reported that older adults exhibit enhanced activity in the left inferior frontal gyrus as well as the superior parietal cortex (12).

Thus, there is need to identify whether more activity in frontal or parietal areas is beneficial for better PM performance or if a decreased activity might be more advantageous. Yet, the mere occurrence of a change in brain activity in concomitance with performance of a behavioural task is not sufficient to confirm a causal relationship between the two phenomena. A functional facilitation and inhibition of different areas via non-invasive brain stimulation would lead to clearer evidence that the neural activity in these areas are not only concomitant with the cognitive operations involved in the behavioural task but actually drives them. Transcranial direct current stimulation (tDCS), a non-invasive technique to selectively facilitate or inhibit excitability of neurons in targeted areas, implemented in a double-blind design such that neither the experimenter nor the participant is aware of the exact stimulation mode may be of help. With tDCS, a facilitation and/or inhibition of the inferior frontal gyrus as well as the superior parietal cortex would help to elucidate further the association between activity in these areas and performance in PM tasks. Thus, tDCS may clarify whether enhancing or inhibiting activity in these areas will lead to a change in PM performance in the elderly and whether this will transfer to performance in attentional control.

3 STUDY OBJECTIVES AND DESIGN

3.1 Hypothesis and primary objective

This study aims to modulate neural activity in the left and right inferior frontal gyrus as well as in the right superior parietal cortex via transcranial direct current stimulation (tDCS). There is evidence from fMRI studies that these areas are involved in PM performance but a causal relation between activity in these areas and responses in PM tasks has not been established so far. The primary objective of this study is the modulation of PM performance via tDCS. We hypothesize that stimulation of the right inferior frontal lobe will lead to changes in PM performance. Whether cathodal or anodal stimulation will enhance performance is not clear yet, since previous fMRI

studies were inconsistent about activity changes in older adults. Further, we expect that anodal stimulation of the left inferior frontal cortex may lead to faster responses to PM stimuli, whereas cathodal stimulation of the same area may lead to prolonged reaction times. Finally, we expect that anodal stimulation of the right superior parietal cortex will lead to better PM performance, whereas cathodal stimulation will have no effect. We also hypothesize that stimulation effects will be transferred to attentional control tasks, as both seem to recruit similar neural structures. We will correct for multiple comparisons, using the Bonferroni- Holm procedure where appropriate.

3.2 Primary and secondary endpoints

The primary endpoint is PM performance as assessed with laboratory tasks; that is, accuracy and reaction times. As secondary endpoints we will use performance in attentional control tasks, performance in naturalistic PM tasks as well as self-rated PM functioning as assessed with PM questionnaires.

3.3 Study design

This is a monocentric study at the University Clinic of Old Age Psychiatry and Psychotherapy and the University of Bern. We will apply a double blind, sham-controlled, and parallel group design. All participants will attend two sessions. The procedures of the sessions will be identical but only during the second session, non-invasive brain stimulation will be applied. Participants will be randomly assigned to one of seven stimulation groups (cathodal stimulation of the right or left inferior frontal gyrus, cathodal or anodal stimulation of the superior parietal cortex, or sham stimulation). The double-blind design will preclude an experimenter as well as a participant bias towards tDCS. In order to control for the successful blinding, both experimenter and participant will be asked to judge whether sham or real anodal tDCS was applied after the stimulation. The subjective assessments will be tested against the actual stimulation group assignment after unblinding subsequently to the final data collection. One of the greatest advantages of tDCS is the existence of a reliable sham stimulation, which involves a ramping up of the current to the intensity used in the real anodal stimulation and switching it off after 30 s. This sham procedure produces the same sensations as real stimulation but without exerting any stimulation effects. While participants will be informed that two conditions (sham and real anodal tDCS) exist, it is necessary to blind both the participant and the experimenter to the actual stimulation in order to avoid effects that are solely attributable to expectations.

<u>Methods to minimise bias:</u>
Randomisation:
We will randomly assign participants to one out of seven groups. We will stratify participants according to 2 variables: age and gender; aiming for a preferable equilibrated gender and age ratio across groups. The participant codes for all groups will be assigned via REDCap implementation, with professional assistance from the Clinical Trials Unit (CTU) in Bern. The implementation of REDCap will allow stratified randomization into groups with equal probability. More precisely, each new participant will be automatically assigned to one the groups via REDCap and this automatic assignment will be done according to stratified randomization. REDCap also allows to provide a numerical code for participants of the behavioural study (see below), which is required for tDCS.

Blinding procedures:
We will apply double-blinding. A numerical code will be randomly assigned to each participant. These codes are provided by the manufacturer of the DC-Stimulator, coding either for real or sham stimulation. The codes will be implemented in REDCap and each new participant of the behavioural study will receive the respective code during randomization. The respective keys will be kept locked up until data acquisition is completed. Thus, participants as well as the examiner will not be aware of group assignment. However, participants will be informed about the existence of the two conditions (i.e., sham and real tDCS).

Unblinding procedures:
Unblinding will only occur after all data is acquired. In case the codes need to be broken at the stage of data collection, this will be done by the PI, who is not involved in data collection or data analysis. This will ensure the blinding of the investigators during the entire process of data collection.

Other methods of minimising bias:
All questionnaires employed in the course of the study have been validated to ensure a good reproducibility.

3.4. Study intervention

As tDCS is a non-invasive technique, no surgical procedures will be used. Surface electrodes will be attached as described below. During real stimulation, 1 mA high-definition transcranial direct current stimulation (HD-tDCS) will be applied for 20 min after a ramp-up phase of 20s. The stimulation period will be completed by a 20s ramp-down phase. We will use 4x1 ring electrodes for HD-tDCS. In this approach, a centre ring electrode (anode or cathode) overlying the target cortical region is surrounded by four return electrodes, which help circumscribe the area of stimulation. This method appears well-tolerated and safe with effective sham-control in older adults for up to 3 mA (13). For right and left inferior frontal gyrus stimulation, the central electrode will be placed over FC6 or FC5 according to the 10-20 system. For the right superior parietal gyrus, the central electrode will be placed over P4. The adjoining electrodes will be placed 3.5 cm away from the central electrode.

HD-TDCS will be applied with a stimulator that delivers direct current with an intensity of 1 mA. Impedance and voltage will be monitored and maintained < 6 kΩ and < 6 V, respectively, across the stimulation. Thus, total current density will not exceed 0.03 mA/cm2 at any point in time and thus will remain below safety limits. The stimulator will be operated in 'study mode', such that neither the participant nor the examiner will be aware of the experimental condition (i.e., double-blind study).

In case of sham stimulation (i.e., control intervention), the electrode positions and the attachment procedures correspond to those of real tDCS but the electrical current will only be ramped up to 1 mA and switched off completely after 30 s of stimulation. The manufacturer (i.e., Soterix) provides an automatic sham feature that is engaged with a simple switch. Auto-sham automatically calculates and produces a sham waveform based on the indicated "real" waveform. For example, for a corresponding real waveform of 1.0 mA and 10 minutes, auto-sham will provide a ramp up/down to 1.0 mA at the start of stimulation, and again after 10 minutes, with the timer automatically adjusted such that the total run time is exactly matched to the real case.

At the end of the experiments, the participants and the experimenter will have to guess which stimulation condition they completed and we will assess possible side effects in conformity with the questionnaire proposed by Brunoni (14).

If not in use or connected to an electric socket for recharging, the stimulator will be stored in a padded case provided by the manufacturer and locked up according to standard procedures.

4 STUDY POPULATION AND STUDY PROCEDURES

4.1 Inclusion and exclusion criteria, justification of study population

We conducted a sample size calculation using g*power. Since there is currently no published HD-tDCS study in PM, we based calculations on a previous TMS study in healthy young individuals, applying event-based PM. The effect size in this study (η^2= 0.32) suggests a required sample size of n=93 to detect robust effects in an ANOVA fixed-effects design with seven groups (i.e., cathodal and anodal stimulation to the right and left inferior frontal gyrus, cathodal and anodal stimulation

to the right superior parietal cortex, sham stimulation). We will increase the sample size to n=15 in each group and will, therefore, include a minimum of n=105 healthy elderly individuals in this project. Prior to enrolment, all participants will be screened over the telephone and will only be invited to the study if deemed eligible.
The participants should be between 60-80 years of age, fluent in German, non-smokers, with normal or corrected-to-normal vision and no severe current of lifetime psychiatric or neurological disorders. Further exclusion criteria will be past head injuries, any history of seizures, current psychotropic medication, dermatosis, metal implants in the head area, current or lifetime alcohol abuse, brain damage, as well as magnetisable implants (e.g., Cardiac pacemakers, brain stimulator). Participants will be included if their Montreal Cognitive Assessment (MoCA) score is ≥ 26 and their Geriatric Depression Score (GDS) is ≤ 6. To ensure comparable verbal intelligence scores, participants will complete a German vocabulary test (Wortschatztest, WST) during the first on-site visit. All participants will give written informed consent prior to testing.

4.2 Recruitment, screening and informed consent procedure

The participants will be recruited via local newspaper advertisements and flyers circulated in Bern. Interested individuals will be provided with a telephone number and Email-address, where they can request more information regarding the research project. Prospective participants will undergo an initial telephone screening as to the eligibility criteria. The investigators will explain to each participant the nature of the study, its purpose, the procedures involved, the expected duration, the potential risks and benefits and any discomfort it may entail. Each participant will be informed that the participation in the study is voluntary and that he or she may withdraw from the study at any time.
All participants will be provided a participant information sheet and two consent forms describing the study and providing sufficient information to make an informed decision about their participation in the study and about the further use of their data. The formal consents of a participant, using the approved consent forms, will be obtained before the participant is submitted to any study procedure. The consent forms will be signed and dated by the investigator or his designee and the participant. A copy of the signed informed consents will be given to the study participant. The consent forms will be retained as part of the study records.
Participants will be informed that they will receive 50, - CHF for their participation.

4.3 Study procedures

All healthy volunteers will participate in a baseline and a stimulation session (one week apart, see Figure 1). The participants will perform the tasks while sitting in front of a 14-inch computer screen in a well-lit, quiet room. At the beginning of each session, the participants will be asked to report the hours of sleep during the last night and to rate the quality of sleep as well as their current concentration (on a scale ranging from 0 to 10).

Figure 1. Study procedures.

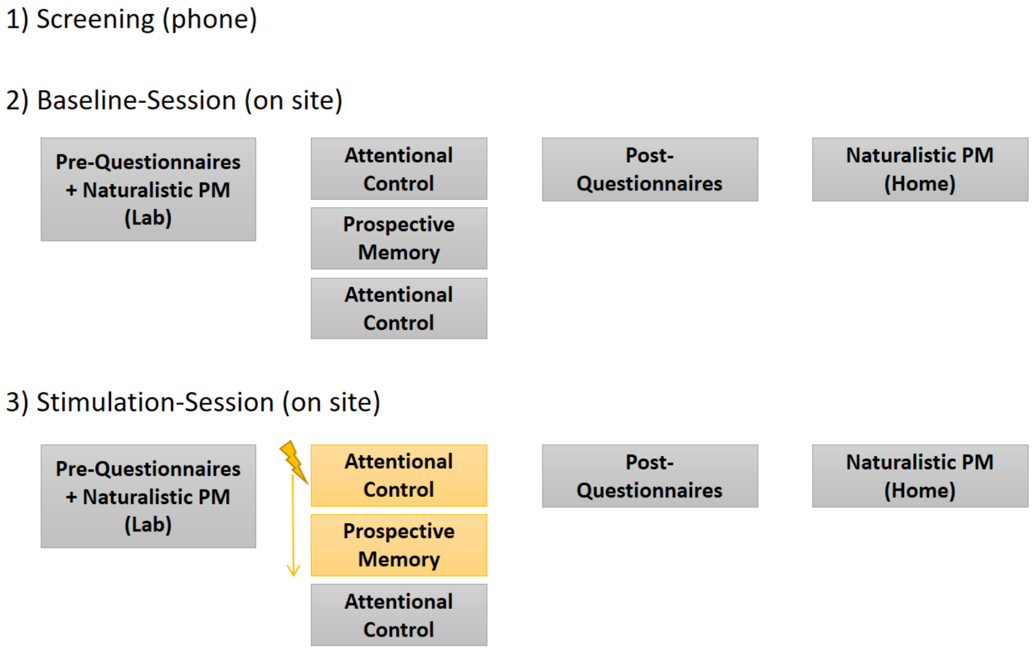

Telephone screening

In the telephone screening prior to the on-site visit, prospective participants will be asked for demographical data and possible contraindications regarding study inclusion. The participants will also receive information regarding the study, a short overview of the expected study time frame and the appointment for the on-site visit will be scheduled (see also Appendix).

On-site visit

On the day of the first on-site visit (i.e., baseline session), participants will read and sign the participant information and consent form. We will then apply cognitive testing using MoCA (15), apply a German vocabulary test for assessing verbal intelligence (WST) (16), and ask the participants to report the hours of sleep during the last night as well as to rate the quality of sleep and their current concentration (on a scale ranging from 0 to 10). To rule out any major depressive symptoms, we will apply the Geriatric Depression Scale (GDS) (17). Further, we will apply a real-life PM task and participants will be asked to fill in a PM questionnaire. Afterwards, we will apply the control and the laboratory PM task. The second session (i.e., stimulation session) will be identical to the first session with the only exception that we will apply tDCS for 20 minutes during the attentional control task and the PM task. We will start the stimulation simultaneously with the first block of the control task and continue stimulation during the PM task. Sham stimulation will last only for a few seconds and will be terminated before the start of the task.
At the end of the experiment, we will we enquire about perceived side effects of the stimulation and control the consistent blinding of the participants with respect to the stimulus condition. Finally, we will apply a real-life PM task that they are intended to perform immediately when returning home and at a specific time-point some days later.

Montreal Cognitive Assessment (MoCA)
The MoCA is a screening test administered in approximately 10 minutes. It assesses several cognitive domains such as short-term and long-term memory as well as visuospatial abilities and executive functions. The MoCA ranges between 0 and 30 with a score of $\leq$ 26 indicating mild cognitive impairment. Thus, we will include healthy elderly participants if their MoCA score is $\geq$ 26. Please note: If baseline assessment in healthy participants indicates cognitive impairment, we will suggest a thorough cognitive assessment at the memory clinic in Bern. In such a case, the prospective participant cannot enter the study.

Verbal Intelligence Scale (Wortschatztest, WST)
The WST measures crystallized intelligence. In this test, participants need to find an existing word in the presence of five distractors within 42 lines of items. The correctly identified words are summarized and converted into an IQ score.

Geriatric Depression Scale (GDS)
The GDS (short version) is a 15-item screening tool used to identify depression in older adults. It is easy to administer and score as it is composed of simple yes/no answers to different questions. It typically takes ~ 5-7 minutes to administer. A score ≥ 6 indicates clinically relevant depressive symptoms.

Prospective memory task
In the PM task, the participants will have to complete blocks of an n-back working memory task (i.e., the ongoing task) and, for some blocks, we will add a PM instruction (see below). We will implement two blocks of three experimental conditions (ongoing-only, event-based PM, and ongoing-only). The second ongoing-only task (i.e., post PM task) serves to measure aftereffects of repeated PM cues by instructing participants that the PM task is completed and they now only need to perform the ongoing task (nevertheless, PM cues will still occur). We will use a 1-back version of an n-back working memory task as the ongoing task, as has been done in prior studies of the collaborating partner (18,19). We will ask the participants to rate pseudorandomized pictures of the Snodgrass and Vanderwart picture set (20). The participants will have to press a ‘yes’ key if the picture was the same as that, which occurred directly before; otherwise, a ‘no’ key needs to be pressed. The ongoing n-back task will consist of 122 trials and will last 10 min. For the PM condition, the participants need to press a target key whenever a picture depicting an animal appears during the n-back task. There will be five PM targets in each block, which will occur at 1:50, 3:50, 5:50, 7:50, and 9:50 min. Every hit on the target key, occurring within 3 s after the presentation of the PM target will be scored as a PM hit. Beside PM target hits, we will compute latency as the median reaction time for correct n-back responses after the exclusion of trials in which a PM target occurred. We will also calculate the PM interference effect as the difference between the latency of the single n-back task and the latency of the n-back items when the PM task was added. We will use only correct n-back responses and exclude PM target trials to avoid finding artificial PM interference effects (21).

Prospective Memory Questionnaires:
We will apply two different questionnaires assessing prospective memory failures, one at each session. These questionnaires serve to allow an application of the colour-pencil task (see below), which measures naturalistic PM. During the baseline session, we will conduct the Prospective Retrospective Memory Questionnaire (PRMQ) (22). The PRMQ contains 16 questions about the frequency of memory failures that have to be answered on a scale ranging from 0 = very often to 4 = never. During the stimulation session, the participants will be asked to fill in the Brief Assessment of Prospective Memory (BAPM) (23). The BAPM assesses the frequency of different everyday PM lapses on a scale ranging from 1 = never to 5 = very often.

Control task:
The control task will assess different aspects of attentional control on a computer with the Attentional Network task (ANT) (24) and different sub-tests of the Test of Attentional Performance (TAP) (25). In one of these sub-tests, the participants will be asked to indicate via button press, which direction an arrow points to. In another subtest, they will need to respond to specific stimuli while ignoring others. In a third subtests, visual and auditory stimuli have to be processed in parallel. Finally, in another subtest, we will apply a set-shifting task during which the participants will need to indicate whether a stimulus appeared left or right to a fixation cross.

Naturalistic PM tasks:
The Royal Prince Alfred Prospective Memory Task (26) consists of four different parts. Part one and two have to be carried out during the session, part three and four at home. There are three parallel versions of the task available; thus, we can use two different versions of the task during the two sessions.
For the Pencil-Colour Task, the participants will be asked to fill in the PM self-rating questionnaires (PRMQ, BAPM) with a blue pencil. They will also be told that whenever a question is labelled as number 5 or 10, they have to fill in this specific question with a red pencil. Both pencils will be placed on the table in front of the participant.

4.4 Withdrawal and discontinuation

Single participants may terminate the study prematurely either in the form of a self-report by the participant or exterior indications. These may relate to the experience of tDCS or discomfort received from any other source. The experiment may also be discontinued for an individual participant based on false statements that led to her/his inclusion in the study. Depending on how far the data acquisition at the time of discontinuation of an individual participant is, the previously acquired data will be crypted after revocation and submitted to the same procedures as complete data sets and evaluated accordingly. Early drop-outs will be replaced by continuing the recruitment of participants into the respective study subpopulation until the designated number of participants is reached and all necessary datasets are acquired.

5 STATISTICS AND METHODOLOGY

5.1. Statistical analysis plan and sample size calculation

For the determination of sample size, we conducted a sample size calculation using g*power. Since there is currently no published tDCS (and neither a HD-tDCS) study in PM, we based calculations on a previous TMS study in healthy young individuals, applying event-based PM (27). The effect size in this study (η^2= 0.32) suggests a required sample size of n=93 to detect robust effects in an ANOVA fixed-effects design with seven groups (i.e., cathodal and anodal stimulation to the right and left inferior frontal gyrus, cathodal and anodal stimulation to the right superior parietal cortex, sham stimulation). We will increase the sample size to n=15 in each group and will, therefore, include n=105 healthy elderly individuals in this project.
The primary objective of this study is the modulation of PM performance via tDCS. We hypothesize that stimulation of the right inferior frontal lobe will lead to changes in PM performance. Whether cathodal or anodal stimulation will enhance performance is not clear yet, since previous fMRI studies were inconsistent about activity changes in older adults. Further, we expect that anodal stimulation of the left inferior frontal cortex may lead to faster responses to PM stimuli, whereas cathodal stimulation of the same area may lead to prolonged reaction times. Finally, we expect that anodal stimulation of the right superior parietal cortex will lead to better PM performance, whereas cathodal stimulation will have no effect. We also hypothesize that stimulation effects will be transferred to attentional control tasks, as both seem to recruit similar neural structures.
For statistical analyses, we will use SPSS (version 21.0; IBM Inc.; USA) and R (version 3.3.1, release 2016; The R Foundation for Statistical Computing: Wien) with R Studio (2016). We will compare the effect of tDCS on PM performance (both laboratory and real-life) and attentional control performance with ANOVAs using stimulation group as between-subject factors and session as a within-subject factor. Since RT typically follow a skewed distribution, a non-parametric equivalent of ANOVA will be used for latency, RT, and PM interference with the nparLD package in R and the same between-subject factors. We will correct for multiple comparisons, using the Bonferroni-Holm procedure (21) where appropriate.

In case data does not meet prerequisites (normal distribution, homogeneity of variance, etc.), non-parametric alternatives to ANOVA will be used instead.

5.2. Handling of missing data and drop-outs

Adequate numbers of participants will be ensured as the recruitment into each study arm will continue until the designated number of participants is reached and all necessary datasets are acquired. Early drop-outs will be replaced by recruitment of new subjects.

6 SAFETY

6.1 Local regulations / Declaration of Helsinki

This study is conducted in compliance with the protocol, the current version of the Declaration of Helsinki, the ICH-GCP, the HRA as well as other locally relevant legal and regulatory requirements.

6.2 Medical Device Category A studies

6.2.1 Definition and Assessment of safety related events

Possible side effects will be documented in the side effect questionnaire at the day of the study. As these are of transient nature, no long-term side effects will be expected. All known contraindications would have led to an exclusion of the participant during the telephone screening.

6.2.2 Pregnancies
N/A

6.3 Reporting to Authorities:
Health hazards that require measures will be reported to the local Ethics Committee via BASEC within 2 days.

6.3.1 Reporting to Sponsor
Health hazards will be reported to the Sponsor within 24 hours upon becoming aware of the event.

6.4 Periodic safety reporting
A yearly safety update-report is submitted by the Investigator to the Ethics Committee via BASEC.

6.7 (Premature) termination of study

The Sponsor-Investigator may terminate the study prematurely according to certain circumstances, e.g.

- Ethical concerns,
- Insufficient participant recruitment,
- When the safety of the participants is doubtful or at risk (e.g. when the benefit-risk assessment is no longer positive),
- Alterations in accepted clinical practice that make the continuation of the study unwise, or
- Early evidence of harm or benefit of the experimental intervention

Upon regular study termination, the Ethics Committee is notified via BASEC within 90 days (ClinO, Art. 38).

Upon premature study termination or study interruption, the Ethics Committee is notified via BASEC within 15 days (ClinO, Art. 38).

A final report will be submitted to the Ethics Committee within one year after finishing (or premature termination).

6.8 Insurance

N/A

7 FURTHER ASPECTS

7.1 Overall ethical considerations

This fundamental research project is conducted as a single centre study with a randomized, placebo-controlled, double-blind design using tDCS to modulate PM performance. We plan on targeting healthy older adults for this project. Results from this project might lead to interventions to counteract cognitive decline in those with a neurodegenerative disorder.
Each participant will be informed that participating the study is voluntary and withdraw from the study at any time will not affect subsequent medical assistance or treatment. Due to the low risk of side effects, the voluntary participation, and the monetary compensation, the disadvantages for the participants seem tenable. All participants will be provided with a participant information sheet describing the study and providing sufficient information to make an informed decision about participating the study.

7.2 Risk-benefit assessment

Participants will undergo tDCS, which is a non-invasive technique to enhance activity of a targeted brain area. Until now, no side effects are known if participants do not carry magnetisable implants in the head area (e.g., deep brain stimulators). The participants will receive financial compensation for their participation (50, - CHF). They might also benefit from tDCS in a way that their PM performance will increase. Due to the low risk of side-effects, the voluntary participation and the monetary compensation, we believe that there is a low risk for the intervention itself. Since participants are only identified by a unique participant number, there is a low risk of unwanted identification of participants. In summary, we believe that our study provides a possible benefit for our participants, while being conducted in a way that provides only minimal risks for participation.

8 QUALITY CONTROL AND DATA PROTECTION

8.1 Quality measures

For quality assurance the sponsor, the Ethics Committee or an independent trial monitor may visit the research sites. Direct access to the source data and all study related files is granted on such occasions. All involved parties keep the participant data strictly confidential.

8.2 Data recording and source data

The CRFs in this trial are implemented electronically using a dedicated electronic data capturing (EDC) system (REDCap). The EDC system is activated for the trial only after successfully passing a formal test procedure. All data entered in the CRFs are stored on a Linux server in a dedicated mySQL database. Responsibility for hosting the EDC system and the database lies within the CTU Bern**.** Paper-pencil data will be stored in lockable drawers (with only investigators knowing the location of the keys), only accessible by authorized personnel. The PIs are responsible and will ensure correct and conscientious use of the data management system.

8.3 Confidentiality and coding

Trial and participant data will be handled with uttermost discretion and is only accessible to authorized personnel who require the data to fulfil their duties within the scope of the study. On the CRFs and other study specific documents, participants are only identified by a unique participant number.
Contact details of the participants (together with an overview of the scheduled appointments and time windows for the separate study parts) will be stored separately from the coding key and only staff members involved in the arrangement of the appointments will have access to this file. The code will only be broken if it is necessary to avert an immediate risk to the health of the person or to guarantee participants rights (e.g. in revoking the consent) or a legal basis exists for breaking the code.

Data security, access and back-up

A list of investigators involved in the project is provided in the study synopsis. Access to the data will be granted only to individuals that require it to conduct the research. For instance, only individuals that need to contact or communicate with participants have access to the participant's identity and contact details. Researchers that do statistical analysis of the study cohort will only get access to pseudonyms. Access is granted through personal accounts (i.e., the CampusID by the University of Bern). Exchange of data is strictly channeled through a system that tracks all changes and prevents permanent deletion of files by researches. The system provides a full audit trail and centralized access control.

Data processing is done on personal computers/laptops and institutional servers. The personal devices are protected against theft/loss by a full system encryption (BitLocker). Communication across the internet is secured through encrypted connections (e.g. SSL or SSH). Regular backups on our local infrastructure provide protection against malicious or accidental data loss.

Referring to RedCAP, the server hosting the EDC system and the database is kept in a locked server-room. Only the system administrators have direct access to the server and back-up tapes. A role concept with personal passwords (site investigator, statistician, monitor, administrator etc.) regulates permission for each user to use the system and database as he/she requires.

All data entered into the CRFs are transferred to the database using Transport Layer Security (TLS) encryption. Each data point has attributes attached to it identifying the user who entered it with the exact time and date. Retrospective alterations of data in the database are recorded in an audit table. Time, table, data field and altered value, and the person are recorded (audit trail).

A multi-level back-up system is implemented. Back-ups of the whole system including the database are run internally several times per day and on external tapes once a day. The back-up tapes are stored in a secure place in a different building.

8.4 Retention and destruction of study data and biological material

All study data are archived for 10 years after study termination or premature termination of the study. At the same time, researchers are requested to delete the data from their local and network storage. In case of paper form, data will be archived in lockable drawers at the research facilities. In case of digital data, the raw data, derived data, code, meta-data, and all history will be exported from the version control system and archived for 10 years on an archival system that stores two copies of the data in two geographically distinct locations. At the end of the project, researchers are asked to remove study data from their devices and local servers. Raw data, code, and all intermediate data can be restored and/or transferred in its entirety or partially.

9 MONITORING AND REGISTRATION

The sponsor will work closely with the investigators at all stages of the study, including the inspection of the study sites for suitability and receiving updates with regard to the progression of the study on a weekly basis. Source data/documents will be accessible to the sponsor and questions will be answered during monitoring.

An independent monitor outside of the study group will be given the authority to monitor the study before, during and after data collection. Source data/documents are accessible to monitors and questions are answered during monitoring.

10. FUNDING / PUBLICATION / DECLARATION OF INTEREST

The study is funded by a grant from the Swiss National Science Foundation awarded to PD Dr. Jessica Peter. There is no conflict of interest. The results of the study will be reported in national and international peer-reviewed journals and at scientific meetings.

10 REFERENCES

1. Common Terminology Criteria for Adverse Events (CTCAE) https://www.eortc.be/services/doc/ctc/CTCAE_4.03_2010-06-14_QuickReference_5x7.pdf
2. Declaration of Helsinki https://www.wma.net/policies-post/wma-declaration-of-helsinki-ethical-principles-for-medical-research-involving-human-subjects/
3. Federal Act on Data Protection (FADP) https://www.admin.ch/opc/en/classified-compilation/19920153/index.html
4. Human Research Act (HRA) https://www.admin.ch/opc/de/classified-compilation/20061313/index.html
5. International Conference on Harmonization (ICH) E6(R2) Guideline for Good Clinical Practice http://www.ich.org/fileadmin/Public_Web_Site/ICH_Products/Guidelines/Efficacy/E6/E6_R2__Step_4_2016_1109.pdf
6. International Conference on Harmonization (ICH) E2A Clinical Safety Data Management: Definitions and Standards for Expedited Reporting http://www.ema.europa.eu/docs/en_GB/document_library/Scientific_guideline/2009/09/WC500002749.pdf
7. Ordinance on Clinical Trials in Human Research (ClinO) https://www.admin.ch/opc/de/classified-compilation/20121176/index.html

1. Ellis J. Prospective Memory: Theory and Applications. In: Brandimonte M, Einstein GO, McDaniel MA, Herausgeber. Prospective memory or the realization of delayed intentions: A conceptual framework for research. New York: Psychology Press; 1996. S. 1–22.
2. Hering A, Kliegel M, Rendell PG, Craik FIM, Rose NS. Prospective memory is a key predictor of functional independence in older adults. Journal of the International Neuropsychological Society. Juli 2018;24(6):640–5.
3. Kliegel M, Martin M, McDaniel MA, Einstein GO. Complex prospective memory and executive control of working memory: A process model. Psychologische Beiträge. 2002;44:303–18.
4. McDaniel MA, Umanath S, Einstein GO, Waldum ER. Dual pathways to prospective remembering. Front Hum Neurosci [Internet]. 14. Juli 2015 [zitiert 13. November 2020];9. Verfügbar unter: http://journal.frontiersin.org/Article/10.3389/fnhum.2015.00392/abstract
5. Ihle A, Kliegel M, Ballhausen N, DaSilva Coelho C, Haas M, Hering A, u. a. Prospective Memory Relates to Attentional Control: Differential Patterns in Old Age. 2019;48(1–2):79–82.

6. Cona G, Scarpazza C, Sartori G, Moscovitch M, Bisiacchi PS. Neural bases of prospective memory: A meta-analysis and the “Attention to Delayed Intention” (AtoDI) model. Neuroscience & Biobehavioral Reviews. Mai 2015;52:21–37.
7. Barban F, Scalici F, Carlesimo GA, Macaluso E, Caltagirone C, Costa A. Medio-lateral functional dissociation of the rostral prefrontal cortex with focal/non-focal cues during a prospective memory task. Brain Imaging and Behavior. August 2020;14(4):1175–86.
8. Gonneaud J, Lecouvey G, Groussard M, Gaubert M, Landeau B, Mézenge F, u. a. Functional dedifferentiation and reduced task-related deactivations underlie the age-related decline of prospective memory. Brain Imaging and Behavior. Dezember 2017;11(6):1873–84.
9. Lamichhane B, McDaniel MA, Waldum ER, Braver TS. Age-related changes in neural mechanisms of prospective memory. Cogn Affect Behav Neurosci. Oktober 2018;18(5):982–99.
10. Magis-Weinberg L, Custers R, Dumontheil I. Sustained and Transient Processes in Event-based Prospective Memory in Adolescence and Adulthood. Journal of Cognitive Neuroscience. 9. Juli 2020;1–22.
11. Peira N, Ziaei M, Persson J. Age differences in brain systems supporting transient and sustained processes involved in prospective memory and working memory. NeuroImage. Januar 2016;125:745–55.
12. Gonneaud J, Kalpouzos G, Bon L, Viader F, Eustache F, Desgranges B. Distinct and shared cognitive functions mediate event- and time-based prospective memory impairment in normal ageing. Memory. 2011;19(4):360–77.
13. Reckow J, Rahman-Filipiak A, Garcia S, Schlaefflin S, Calhoun O, DaSilva AF, u. a. Tolerability and blinding of 4x1 high-definition transcranial direct current stimulation (HD-tDCS) at two and three milliamps. Brain Stimulation. September 2018;11(5):991–7.
14. Brunoni AR, Amadera J, Berbel B, Volz MS, Rizzerio BG, Fregni F. A systematic review on reporting and assessment of adverse effects associated with transcranial direct current stimulation. International Journal of Neuropsychopharmacology. September 2011;14(8):1133–45.
15. Nasreddine ZS, Phillips NA, BÃ©dirian V, Charbonneau S, Whitehead V, Collin I, u. a. The Montreal Cognitive Assessment, MoCA: A Brief Screening Tool For Mild Cognitive Impairment: MOCA: A BRIEF SCREENING TOOL FOR MCI. Journal of the American Geriatrics Society. April 2005;53(4):695–9.
16. Schmidt K, Metzler P. Wortschatztest (WST). Weinheim: Beltz Test GmbH; 1992.
17. Yesavage JA, Sheikh JI. 9/Geriatric Depression Scale (GDS). 1986;5(1– 2):165–73. Clin Gerontol. 1986;5(1–2):165–73.
18. Zinke K, Einert M, Pfennig L, Kliegel M. Plasticity of Executive Control through Task Switching Training in Adolescents. Front Hum Neurosci [Internet]. 2012 [zitiert 15. Januar 2021];6. Verfügbar unter: http://journal.frontiersin.org/article/10.3389/fnhum.2012.00041/abstract
19. Jäger T, Kliegel M. Time-based and event-based prospective memory across adulthood: underlying mechanisms and differential costs on the ongoing task. J Gen Psychol. Januar 2008;135(1):4–22.

20. Snodgrass JG, Vanderwart M. A standardized set of 260 pictures: Norms for name agreement, image agreement, familiarity, and visual complexity. Journal of Experimental Psychology: Human Learning and Memory. 1980;6:174–215.
21. Holm S. A Simple Sequentially Rejective Multiple Test Procedure. Scandinavian Journal of Statistics. 1979;6(2):65–70.
22. Smith G, Della Sala S, Logie RH, Maylor EA. Prospective and retrospective memory in normal ageing and dementia: a questionnaire study. Memory. 2000;8(5):311–21.
23. Man DWK, Fleming J, Hohaus L, Shum D. Development of the Brief Assessment of Prospective Memory (BAPM) for use with traumatic brain injury populations. Neuropsychological Rehabilitation. Dezember 2011;21(6):884–98.
24. Ishigami Y. The Attention Network Test-Interaction (ANT-I): reliability and validity in healthy older adults. Exp Brain Res. 2016;13.
25. Zimmermann P, Fimm B. Testbatterie zur Aufmerksamkeitsprüfung für Kinder (KITAP) Herzogenrath: Psytest; 2004. In: Leclercq M, Zimmermann P, Herausgeber. Applied Neuropsychology of Attention: Theory, Diagnosis and Rehabilitation. Psychology press; 2002. S. 110–51.
26. Radford KA, Lah S, Say MJ, Miller LA. Validation of a New Measure of Prospective Memory: The Royal Prince Alfred Prospective Memory Test. The Clinical Neuropsychologist. Januar 2011;25(1):127–40.
27. Bisiacchi PS, Cona G, Schiff S, Basso D. Modulation of a fronto-parietal network in event-based prospective memory: An rTMS study. Neuropsychologia. Juli 2011;49(8):2225–32.

Appendix 1: Schedule of assessments

	~ -18 to -14 days	0	+ 7 days
**Visit**	**Screening**	**On-site visit 1**	**On-site visit 2**
**Oral patient information**	+		
**Inclusion-/ exclusion criteria**	+	+	
**Demographics**	+		
**Written informed consent**		+	
**Questionnaires (GDS, PANAS, POMS, PM)**		+	+
**Cognition (MoCA, WST)**		+	
**Form of the day (e.g., sleep)**		+	+
**Intervention (tDCS)**			+
**PM task**		+	+
**Control task**		+	+
**Naturalistic PM task**		+	+
**Blinding, side effects**			+
